# hiPSC-derived cardiac fibroblasts dynamically enhance the mechanical function of hiPSC-derived cardiomyocytes on an engineered substrate

**DOI:** 10.3389/fbioe.2025.1546483

**Published:** 2025-05-23

**Authors:** Mitchell Josvai, Jodi Lawson, Harshal Kanade, Meghana Kalluri, Corey L. Anderson, Jianhua Zhang, Alana Stempien, Lee L. Eckhardt, Timothy J. Kamp, Wendy C. Crone

**Affiliations:** ^1^ Department of Biomedical Engineering, University of Wisconsin-Madison, Madison, WI, United States; ^2^ Wisconsin Institute for Discovery, University of Wisconsin-Madison, Madison, WI, United States; ^3^ Department of Medicine, Division of Cardiovascular Medicine, University of Wisconsin-Madison, Madison, WI, United States; ^4^ Department of Cell and Regenerative Biology, University of Wisconsin-Madison, Madison, WI, United States; ^5^ Department of Nuclear Engineering and Engineering Physics, University of Wisconsin-Madison, Madison, WI, United States; ^6^ Department of Mechanical Engineering, University of Wisconsin-Madison, Madison, WI, United States

**Keywords:** hiPSC, cardiomyocytes, cardiac fibroblasts, mechanical function, tissue engineering

## Abstract

**Introduction:**

Cardiac fibroblasts deposit and turnover the extracellular matrix in the heart, as well as secrete soluble factors that play critical roles in development, homeostasis, and disease. Coculture of CFs and human induced pluripotent stem cell (hiPSC)-derived cardiomyocytes (CMs) enhances CM mechanical output, yet the mechanism remains unclear.

**Methods:**

Here, we use an *in vitro* engineered platform to compare the effects on CM mechanical function of direct CM-CF Coculture and soluble signaling alone through CF Conditioned Medium to a CM Only monoculture. Mechanical analysis is performed using digital image correlation and custom software to quantify the coordination and organization of CM contractile behavior.

**Results:**

CM-CF Coculture induces larger CM contractile strains, and an increased rate of spontaneous contraction compared to CM Only. Additionally, CM-CF Cocultures have increased contractile anisotropy and myofibril alignment and faster kinetics. The paracrine effects of fibroblast conditioned medium (FCM) are sufficient to induce larger contractile strains and faster contraction kinetics with these effects remaining after the removal of FCM. However, FCM does not influence CM spontaneous rate, contractile alignment, anisotropy, or relaxation kinetics compared to CM Only control.

**Discussion:**

These data suggest that hiPSC-CFs exert dynamic and multifactorial effects on the mechanical function of hiPSC-CMs and highlight the importance of CFs in both the native heart and *in vitro* cardiac models. Further, this work demonstrates the applicability of the coculture–conditioned medium–monoculture paradigm to decouple the effects of paracrine factor and cell-cell signaling on hiPSC-CM mechanical function and maturation.

## 1 Introduction

Cardiomyocytes (CMs) are the primary contractile cells of the heart, responsible for executing coordinated contractions sufficient to maintain cardiac function (Zhou and Pu, 2016; Litviňuková et al., 2020). Cardiac fibroblasts (CFs) are highly abundant, making up 20%–30% of cells in cardiac tissue (Souders et al., 2009; Litviňuková et al., 2020; Tucker et al., 2020). Functionally, CFs are necessary for maintenance and remodeling the extracellular matrix (ECM), direct cell-to-cell communication, and paracrine signaling factor secretion (MacKenna, 2000; Camelliti et al., 2005; Sadoshima and Weiss, 2014; Harrington and Moore-Morris, 2024). CFs play a role in cardiac development, homeostasis, and repair in response to myocardial injury or disease, though their direct effects on CM mechanical function and maturation remain unclear (Sandanger et al., 2013; Hall et al., 2021; Tian and Ren, 2023).

Human induced pluripotent stem cells (hiPSCs) offer researchers a near inexhaustible source of CMs and CFs for *in vitro* studies (Dhahri et al., 2022). Yet despite recent advances, potential applications and therapies involving hiPSC-derived cardiomyocytes (hiPSC-CMs) are hindered by the relative immaturity of these myocytes in comparison to primary adult CMs (Chen et al., 2009, p. 2; Aigha and Raynaud, 2016; Wu et al., 2023). hiPSC-CMs display distinct differences from adult cells, with electrophysiological, morphological, and mechanical characteristics similar to embryonic CMs (Zhang et al., 2009; Bedada et al., 2014; Denning et al., 2016; Koivumäki et al., 2018; Notbohm et al., 2019). Methods to influence hiPSC-CMs towards a more mature phenotype *in vitro* have been developed, such as chronic mechanical and electrical stimulation (Tulloch et al., 2011; Ma et al., 2018), ionic current enhancement (Vaidyanathan et al., 2016), metabolic or hormonal cues (Horikoshi et al., 2019; Funakoshi et al., 2021; Floy et al., 2022), and coculture with alternative cell types (Dunn et al., 2019; Beauchamp et al., 2020). CM-CF coculture has been shown to increase the amplitude of force generation by CMs as well as the spontaneous rate of contraction (Zhang et al., 2013; Giacomelli et al., 2020; Lock et al., 2024). Additionally, we have previously shown that CFs improve calcium kinetics and increase contractile strain when in coculture with CMs (Napiwocki et al., 2021b; Stempien et al., 2024). Such findings demonstrate the importance of developing models of myocyte-fibroblast-ECM interactions with the goal of understanding the bidirectional communication between cell types, and their role in mechanical function of the cardiac syncytium. However, fibroblast-myocyte communication is both soluble factor- and matrix-mediated, and it has previously been unclear which mechanism underlies the enhancements in CM mechanical output, if not both (Tsuruda et al., 2002; Divakaran et al., 2009; Goldsmith et al., 2014; Israeli-Rosenberg et al., 2014).

The selection of culture model is crucial in determining the biological and physiological relevance of *in vitro* findings. Conventional 2-dimensional (2D) monoculture and coculture monolayer models are simple to fabricate and use but lack complex architectural and organizational features found in the native tissue (Chilton et al., 2007; Rother et al., 2015; Belanger et al., 2023). Additionally, CFs cultured on stiff surfaces, such as glass or conventional tissue culture plastic (TCP) may undergo a rapid transition to myofibroblasts, which are increasingly implicated in disease pathogenesis (Santiago et al., 2010; D’Urso and Kurniawan, 2020; Hall et al., 2023; Felisbino et al., 2024). 2D models which incorporate physiologic substrate stiffness, micropatterned ECM, electrospun fibers, or other topographical cues represent an increase in complexity and influence myocyte phenotype, structure, and function beyond what can be achieved in traditional monolayer systems (Kaji et al., 2011; Liu et al., 2017; Napiwocki et al., 2021a). Further, these systems enable studying of the direct matrix and indirect paracrine effects of fibroblast-myocyte communication but are insufficient to separate the distinct mechanisms.

Transwell and conditioned medium approaches have previously been used to determine the effects of soluble paracrine factors originating in one cellular phenotype on another phenotype (Fredj et al., 2005; Stone et al., 2019; Zhou et al., 2019; Guo et al., 2023). Studies with conditioned media have demonstrated that fibroblasts mediate cardiomyocyte hypertrophy through the promotion of angiotensin II (Gray, 1998; Bhullar and Dhalla, 2022). Other groups have shown that cardiac fibroblast conditioned media (FCM) can induce CM differentiation in cardiac progenitor cells and hiPSCs through fibroblast growth factor (FGF) signaling (Chan et al., 2010; Zhang et al., 2014). Studies with primary rat CMs show that FCM alters ion channel expression and induces CM hypertrophy (LaFramboise et al., 2007; Pedrotty et al., 2009). A clear relationship exists between soluble factor-mediated CF signaling and CM phenotype. However, these studies were primarily performed in simplified non-human systems, and did not properly prevent against CF to myofibroblast transition, which is known to have deleterious effects on pathophysiology. As a result, the impacts of hiPSC-CF paracrine signaling on the contractile function of hiPSC-CMs remains to be explored.

This work explores the differential effects of CM-CF coculture and CF conditioned medium on hiPSC-CM mechanical function on an engineered 2D platform. We aim to determine how coculture and conditioned media influence CM mechanical function and whether these effects stem from ECM remodeling and direct CF contact, CF-mediated paracrine signaling, or an interplay of the two. Given the role of paracrine signaling in heart development, we hypothesize that conditioned media will reveal the impact of CF-mediated soluble and matrix signaling on CM function. These changes may reflect functional maturation, offering a simpler, cost-effective alternative to methods such as RNAseq or mass spectrometry for assessing CM maturation. Building on prior work, we enhance our image analysis software to quantify single cell and sarcomere structures and extend DIC analysis to measure complex strain fields and contraction kinetics. Notably, we also highlight the role of soluble paracrine signaling between CMs and CFs in this engineered *in vitro* model, alongside previously reported ECM mechanisms.

## 2 Materials and methods

### 2.1 hiPSC culture and maintenance

The male hiPSC line DF19-9-11T (WiCell, Madison, WI) displaying a normal karyotype was used for both CM and CF differentiation (Acevedo-Acevedo and Crone, 2015; The International Stem Cell Initiative et al., 2018). hiPSCs were thawed and maintained on Matrigel (GFR, Corning, Corning, NY) coated plates (8.7 μg/cm^2^) in mTesR1 medium (WiCell). hiPSCs were dissociated with Versene and passaged in mTesR1 supplemented with 10 μM ROCK inhibitor (Y-27632, Tocris, Bristol, United Kingdom) at a lower density after reaching 50%–60% confluency. Multiple passages were performed prior to CM or CF differentiation to ensure homogeneity in the hiPSC culture. hiPSCs were between passages of 40 and 60 at the initiation of differentiation. *Mycoplasma* screen was routinely performed in the cell culture incubators.

### 2.2 CM differentiation, purification, and culture

hiPSCs were differentiated to hiPSC-CMs using the small molecule GiWi method (Lian et al., 2012; 2013) and purified using lactate medium (Tohyama et al., 2013). Briefly, hiPSCs were seeded on Matrigel coated plates in mTesR1 with 10 μM ROCK inhibitor for 5 days or until 100% confluent. On day 0 of differentiation, cells were treated with 12 μM of the GSK3 inhibitor CHIR 99021 (Biogems, Westlake Village, CA) in RPMI 1640 (Thermo Fisher Scientific, Walthem, MA) medium with 2% B27 minus insulin supplement (Thermo Fisher Scientific). Twnty-four hours after the addition of CHIR (day 1), the medium was changed to RPMI 1640 with 2% B27 minus insulin supplement without CHIR. 72 h after the addition of CHIR (day 3), the medium was changed to RPMI 1640 with B27 minus insulin and 5 μM of the Wnt inhibitor IWP2 (StemGent, Beltsville, MD). On days 5 and 7, cells were cultured with RPMI 1640 with B27 minus insulin. From days 9–15, cell were cultured with RPMI 1640 with 2% B27 complete supplement (Thermo Fisher Scientific) with medium change every other day. Beating cells typically were observed starting between days 10 and 13 of differentiation. Day 15 differentiated cells were measured by flow cytometry for cTnT^+^ cells (Supplementary Figure S1). Day 15 hiPSC-CMs were dissociated with TrypLE 10× (Thermo Fischer) for 10 min and cryopreserved in 90% fetal bovine serum (FBS, Invitrogen, Waltham, MA) with 10% DMSO (Sigma, St. Louis, MO). Cryopreservation was performed by storing CMs in a Mr. Frosty Freezing Container (Thermo Fischer) overnight at −80°C before transferring to liquid nitrogen for storage. Day 15 cryopreserved hiPSC-CMs were thawed on Matrigel-coated 6-well plate at a density of 2.5 million cells/well in EB20 medium: DMEM/F12 (Life Technologies, Carlsbad, CA), 20% FBS, 1% NEAA (Life Technologies), 0.5% GlutaMax (Life Technologies) and 7 μM 2-Mercaptoethanol (Sigma). In summary, the cryopreserved cells (CMs) were thawed by manually warming the vial in a 37°C water bath until only a small ice crystal remained. Subsequently, the vial contents were transferred to a conical tube, and 11 mL of room temperature EB20 medium was gradually added dropwise. CMs were then centrifuged for 5 min at 1,000 rpm before resuspending in EB20 and plating on Matrigel-coated six-well plate. A minimum of five unique iPSC-CM differentiations were used for CM Only, CF Conditioned, and CM-CF Coculture. Three unique differentiations were used for the CF Conditioned treatment group.

Two days after thaw, hiPSC-CMs were purified using lactate medium: RPMI 1640 no glucose (Life Technologies), 2% B27 complete, and 5 mM lactate (Sigma) (Tohyama et al., 2013). Lactate medium was changed every other day for 10 days, and then changed to EB2 medium on day 27: DMEM/F12 (Life Technologies), 2% FBS (Life Technologies), 1% NEAA (Thermo Fisher Scientific), 0.5% GlutaMax (Life Technologies) and 7 μM 2-Mercaptoethanol (Sigma), which was replaced every 2 days thereafter. Flow cytometry confirmed that after differentiation and lactate purification, hiPSC-CM purities of greater than 95% were achieved (Napiwocki et al., 2021b).

### 2.3 CF differentiation and culture

hiPSCs were differentiated hiPSC-CFs as described previously (Zhang et al., 2019). hiPSCs were dissociated and seeded on Matrigel-coated six-well plates at the density of 2 × 10^6^ cells/well in mTeSR1 medium supplemented with 10 μM ROCK inhibitor. Cells were cultured for 5 days in mTeSR1 medium with medium change daily until 100% confluence was reached. On day 0 of differentiation, medium was changed to 2.5 mL RPMI with 2% B27 minus insulin and 12 μM CHIR. At day 1 (24 h after CHIR added), medium was changed to 2.5 mL RPMI plus 2% B27 minus insulin. At day 2, the medium was changed to 2.5 mL of the defined fibroblast culture medium (CFBM): DMEM high glucose (Thermo Fischer), 1.75% GlutaMAX Supplement (Thermo Fischer), 0.25% HLL supplement (Millipore), 0.1% Ascrobic Acid (Millipore), 0.1% Hydrocortisone Hemisuccinate (Millipore), and 0.1% Rh Insulin (Millipore), supplemented with 75 ng/mL basic Fibroblast Growth Factor (bFGF; WiCell). Cells were fed every other day with CFBM supplemented with 75 ng/mL bFGF and cultured until day 20. hiPSC-CFs were maintained in FibroGRO (Millipore EMD, Burlington, MA) plus 2% FBS on tissue culture plastic. By passage 3, CFs were 99% TE-7^+^ (Supplementary Figure S1), a marker for human CFs (Zhang et al., 2019; Napiwocki et al., 2021a). Cryopreserved hiPSC-CFs were thawed in FibroGRO plus 2% FBS and plated onto six-well tissue culture plastic plates at a density of 55,100 cells/well in 2 mL of FibroGRO medium plus 2% FBS. hiPSC-CFs were maintained in FibroGRO plus 2% FBS medium which was replaced every 2 days. hiPSC-CFs were passaged at least once after thawing before use in experiments.

### 2.4 Substrate fabrication and ECM patterning

To mimic the 10 kPa elastic modulus of the healthy heart, compliant PDMS substrates for cell seeding were fabricated by blending Sylgard 184 and Sylgard 527 (Dow Corning Corporation, Auburn, MI) in defined ratios (Berry et al., 2006; Palchesko et al., 2012). Sylgard 184 was prepared by combining 10 parts base to 1 part cure and mixing with a glass stir rod for 5 min. Sylgard 527 was prepared by combining equal amounts of volumes A and B and mixing for 5 min. To generate 10 kPa substrates, Sylgard 184 and Sylgard 527 were mixed in a predetermined 52:1 ratio for 5 min and poured into a 100 mm Petri dish. The uncured PDMS was placed under a vacuum for 20 min to remove gas impurities. The dish was removed from the vacuum and cured for 12 h at 60°C before being cut to the desired dimensions using a razor blade. Mechanical analysis was previously performed to ensure that the desired PDMS modulus was achieved using the 52:1 ratio (Napiwocki et al., 2017). The compliant substrates were attached to either a 6- or 12-well plate (Corning, Corning, NY) using a drop of Sylgard 184 and curing for 6 h at 60°C. Samples were subjected to 10 min UV exposure to sterilize before patterning.

Microcontact printing and soft lithography were used to generate compliant substrates with patterned ECM proteins in defined geometries as previously described (Salick et al., 2014; Napiwocki et al., 2021a; Josvai et al., 2024). A master Si wafer (FlowJEM, Toronto, ON, Canada) was used to generate reusable PDMS stamps. Sylgard 184 PDMS was cured for 6 h at 60°C before being cut into individual stamps for micropatterning. The stamps were coated with Matrigel and incubated at 37°C overnight before removing excess Matrigel. Stamps were gently dried with a nitrogen airstream to remove residual moisture without dislodging the attached proteins. A polyvinyl alcohol (PVA) film was produced by mixing 0.5 g PVA beads (Sigma) with 10 mL deionized water for 20 min at 100°C. The PVA solution was poured into a 100 mm Petri dish and left uncovered to dry overnight. The dried film was cut into sections with an area slightly larger than the PDMS stamps and brought in contact with the dry stamps that had previously been coated in Matrigel. The patterned Matrigel was allowed to transfer to the PVA film for 1 h at 37°C (Yu et al., 2012). Transfer efficiency was increased by placing a glass slide and a 50 g weight on top of each stamp. After incubation, the PVA film was removed from the stamp and brought in contact with the PDMS substrates. The Matrigel was allowed to transfer to the substrate for 20 min at 37°C before being washed with 3 mL phosphate-buffered saline (PBS) to dissolve the PVA film. After 10 min, an additional PBS wash step was performed, removing the soluble PVA film and leaving only the patterned ECM proteins on the 10 kPa substrates. Substrates were maintained briefly in PBS until cell seeding occurred.

### 2.5 Cell seeding on patterned substrates

Day 30 purified hiPSC-CMs were dissociated and singularized with TrypLE 10X (Life Technologies) for 12 min and centrifuged for 5 min at 1,000 rpm. CMs were resuspended in EB20 medium and seeded onto patterned substrates at a density of 2,500 CMs/mm2 (90,000 CMs/35.605 mm^2^). The day of seeding is referred to as day 0 of experimental culture. CM Only and fibroblast conditioned medium (CF Conditioned) samples were seeded with purified hiPSC-CMs at a density of 2,500 CMs/mm2. CM-CF coculture samples were seeded at an equivalent CM density, along with a 10:1 ratio of hiPSC-CFs (Stempien et al., 2024). The 10:1 ratio is utilized as it enables proliferative hiPSC-CFs to populate most of the initially unoccupied areas in the pattern by day 18 (Figure 2A), while preventing the culture from being dominated by hiPSC-CFs, which would reduce physiological relevance due to an atypically large CF:CM ratio. hiPSC-CFs were treated with TrypLE Express (Life Technologies) for 3 min, centrifuged for 5 min at 1,000 rpm, and seeded onto patterned PDMS cocultures using EB20 medium at a density of 250 hiPSC-CFs/mm2 (9,000 CFs/35.605 mm^2^). A 1 cm fluorinated ethylene propylene (FEP) tube was placed on top of the PDMS substrates and the cell suspension was added inside the tube to control attachment region. The FEP tube was removed after 24 h and an additional 1.5 mL of EB20 medium was added. 48 h after seeding (day 2, 24 h after seeding chamber removal), the medium was changed to EB2 medium and exchanged every 2 days during the length of culture. CM only and CM-CF coculture samples were maintained with only basal EB2 medium from days 2–18, while FCM samples were maintained with fibroblast conditioned EB2 from day 2 until the end of experimental culture (described below).

### 2.6 Transient CF conditioned medium experiment

**FIGURE 1:**
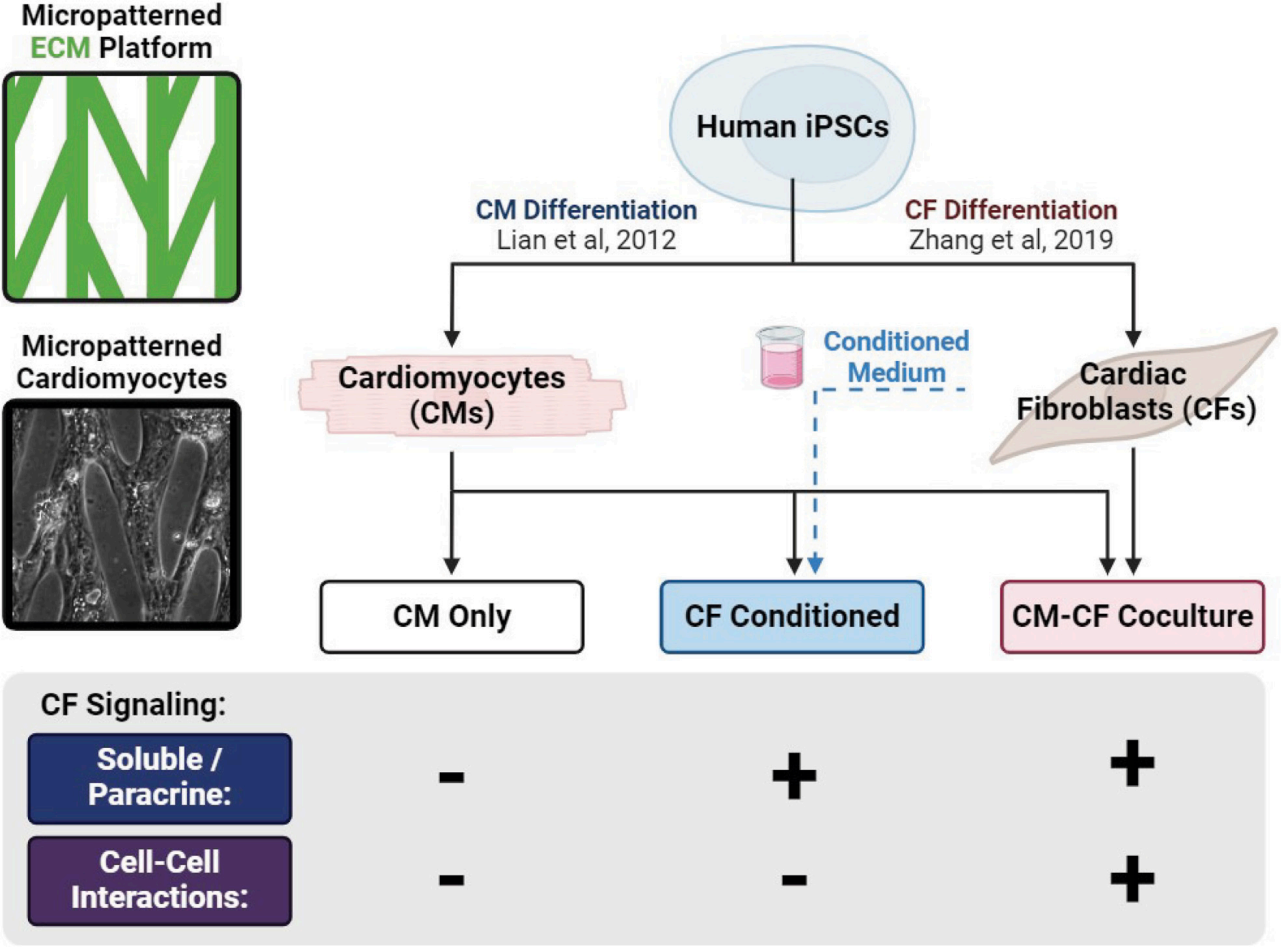
Graphical representation of experimental culture groups. hiPSCs are differentiated into separate populations of CMs and CFs and split among three experimental groups: CM monocultures supplied with basal medium (CM Only), CM monocultures supplied with medium conditioned by a CF monoculture (CF Conditioned), and cocultures of CMs and CFs together (CM-CF Coculture).

For CF conditioned samples, EB2 medium was conditioned by an unpattterned CF monoculture, as described below, before being transferred to the samples unaltered (for CF Conditioned) or at a 1:1 dilution with fresh EB2 (for 0.5 CF Conditioned, results provided in supplemental). On day 0 of experimental culture, when CM Only, CF Conditioned, and CM-CF coculture samples were seeded onto patterned substrates, hiPSC-CFs were treated with TrypLE Express for 3 min, centrifuged for 5 min at 1,000 rpm, and seeded onto 10 kPa unpatterned Matrigel monolayers in EB20 medium at a density of 95 hiPSC-CFs/mm2 (9,000 CFs/96 mm^2^). The timing and absolute number of hiPSC-CFs during seeding was equal for CF Conditioned and CM-CF coculture samples to ensure similar concentrations of paracrine factors, and CF monoculture samples were cultured on 10 kPa PDMS to prevent myofibroblast transition in these samples. Every other day, CF conditioned EB2 was removed from CF monocultures and supplied to FCM samples unaltered or diluted 1:1 with basal EB2. CF monocultures were then fed with fresh EB2. A graphical depiction of the experimental culture conditions is provided as Figure 1.

For the transient CF conditioned medium experiment, no CFs were present and CF Conditioned medium was provided to the patterned CM cultures from days 2 until 10. From days 12 until 18, these samples were provided with basal (unconditioned) EB2 in the same manner as CM Only samples. No medium change occurred on day 11.

### 2.7 Brightfield microscopy and strain quantification

**FIGURE 2:**
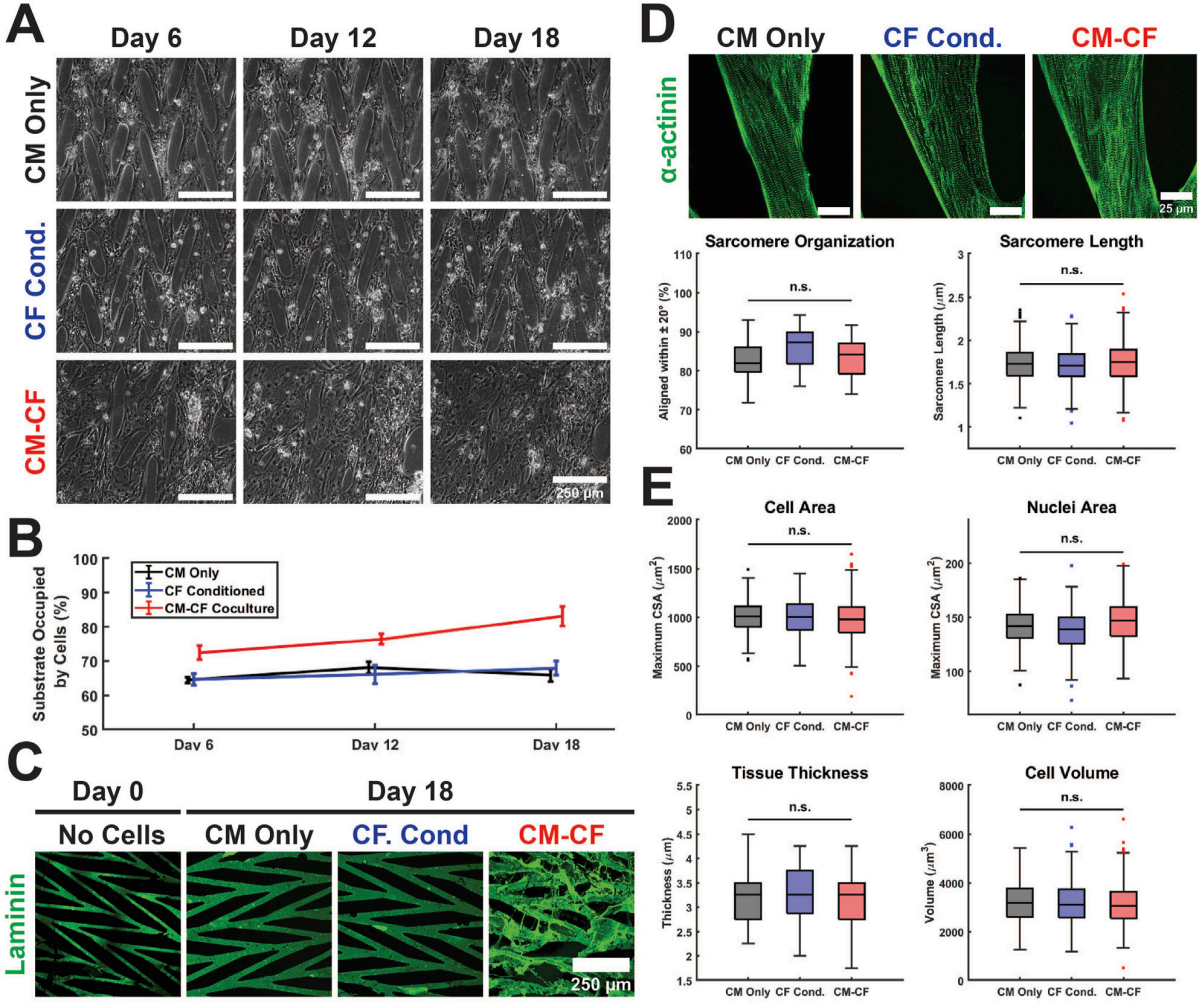
Culture conditions do not alter hiPSC-CM structural characteristics on a micropatterned platform. **(A)** Representative Day 6 (left), Day 12 (center column), and Day 18 (right) images of CM Only (top), CF Conditioned (center row), and CM-CF Coculture (bottom) groups. Scale bars = 250 µm. **(B)** CM-CF Cocultures demonstrate progressive remodeling and increases in substrate occupation, while CM Only and CF Conditioned remain unchanged. **(C)** Representative images of day 0 (right) and day 18 (left) samples stained for the ECM component laminin (green). Day 0 samples are prior to cell seeding and represent only the micropatterned ECM and no cellular modifications. Scale bar = 250 µm. **(D)** Representative images stained for sarcomeric alpha actinin (top). The day 18 properties of the cardiac sarcomere, including length and organization, are unaltered across conditions (bottom). Scale bars = 25 µm. N = 20 (5 samples, 4 locations each). **(E)** No changes are observed in the day 18 cell area, nuclei area, tissue thickness, or cell volume across conditions. N = 20 (5 samples, 4 locations each). A minimum of 5 unique hiPSC-CM differentiations were used for each experimental group.

Phase contrast brightfield microscopy videos were acquired of the cells’ spontaneous contractions on days 6,12, and 18 using a Nikon Eclipse Ti microscope with a Plan Fluor 10x NA 0.3 objective and Nikon DS-QiMc camera with samples maintained at 37°C as we have described previously (Stempien et al., 2022). At least two contractions or a maximum of 400 frames were acquired for each sample with an acquisition rate of 18.92 fps. Mechanical analysis was performed using previously developed open-source digital image correlation (DIC) software, with custom add-ons and modifications (Notbohm et al., 2019; Napiwocki et al., 2021a; Stempien et al., 2022). The random high contrast pattern from the phase contrast of the cells is sufficient to allow tracking of displacements using Fast Iterative Digital Image Correlation (FIDIC) (Bar-Kochba et al., 2015). DIC is advantageous as it does not require the addition of exogenous speckle patterns to the culture system and may be performed at multiple experimental timepoints. Each frame was analyzed relative to the first frame which contained cells in a relaxed state. Full field 2D displacements, Ux and Uy, were computed for each frame using the input parameters of a target subset size of 48 pixels (31.2 μm) and subset spacing of 12 pixels (7.8 μm). From the displacements, x and y strains were calculated by taking the gradient of the displacement. Principal strains, ε_1_ and ε_2_, were computed for each time point. A binary mask was created using edge detection techniques to eliminate data from areas not occupied by cells (Treloar and Simpson, 2013). The masks used to define regions of cell occupancy for DIC analysis were also used to quantify occupancy in the bright field images of Figure 2A, as described previously (Josvai et al., 2024). Second principal strain (i.e., contractile strain, ε_2_) was the primary metric of mechanical function. Full field contractile strain values were averaged for each frame of the video and the maximum was used as the metric for comparison between sample conditions and for kinetic analysis. The spontaneous rate was calculated using the distances between the peaks of contraction in seconds and converted to BPM using the peak-to-peak method described in Stempien et al., 2024; Stempien et al., 2024) Importantly, the spontaneous rate and the amplitude of contraction were calculated from the same video acquisitions, thus the comparison in Supplementary Figure S5 is valid. Only videos which contained multiple contractions and could calculate the spontaneous rate were included in the strain, contraction rate, displacement trajectory alignment, and contractile kinetics datasets.

### 2.8 Displacement trajectory quantification

The alignment of displacement trajectories of contracting cells was computed to quantify the organization of contraction behavior. The total vertical and horizontal displacements for each location at each peak of contraction were used to compute the angle and magnitude of the resultant vector through the four-quadrant inverse tangent (MATLAB atan2d function). Trajectories with small magnitudes, likely occurring in regions of the pattern where no cells were present, were filtered by eliminating values below a threshold of 0.75 of the median trajectory magnitude for that sample. To quantify directionality, the predominant principal strain orientation identified by DIC software was identified (the mode of all displacement vector trajectories rounded to the nearest whole integer, Supplementary Figure S3). The percentage of displacement trajectories at the peak of contraction aligned within 20 degrees of the predominant strain orientation was used as a metric of contractile organization. The circular variance was adapted in 2D from Lee et al. (Lee et al., 2020) and calculated using a modified version of the open-source MATLAB package CircStat v.1.21.0.0 (Berens, 2009). Weighted anisotropy was defined as the sum of the magnitudes of all displacement trajectories aligned within 20 degrees of the predominant angle divided by the sum of the magnitudes of all displacement trajectories with orientations not aligned within 20 degrees of the predominant angle.

### 2.9 Contractile kinetics analysis

Custom Python software (available at: github. com/harshalkanade) was used to analyze strain kinetics. Initiation, each file was filtered to remove noise and better identify individual contraction events. All kinetics results have ∼50 ms resolution resulting from the capabilities of the imaging system. Cell area at strain threshold was calculated by determining the pixel area above a 0.008% strain threshold value as a percentage of the full area occupied by cells (not masked during strain calculations). The time at peak contraction was calculated by normalizing contraction behavior and determining the number of frames between the intercepts of strain behavior and a 95% normalized strain threshold. For kinetics analyses, the intercepts of the 90% normalized strain threshold were calculated and used as the 90% contraction and 10% relaxation points. From the 10% relaxation point (intercept of relaxation and 90% normalized strain), the instantaneous slope was calculated for each subsequent frame until slope decreased below a threshold of 0.1%/frame, where the contraction event had ended. The 90% relaxation point was used for calculations to remove any noise in determining the absolute relaxation frame. The time between the 10% and 90% relaxation points was used as the relaxation time, with the slope between these points used as the relaxation rate. Likewise, the upstroke rate was calculated as the slope between the 10% and 90% contraction strain indices. Similar to cardiac action potentials, upstroke contraction behavior occurred much more quickly than relaxation behavior, as such the upstroke time was not analyzed due to resolution limitations.

### 2.10 Immunocytochemical labeling and imaging

Samples were washed once with PBS and fixed in 4% paraformaldehyde (PFA; Electron Microscopy Sciences, Harfield, PA) for 15 min at room temperature and then washed with PBS. Cells were permeabilized with 0.1% Triton-X 100 (Sigma-Aldrich) in a blocking solution containing 5% goat serum in PBS for 1 h. Primary antibodies were diluted in the blocking solution and incubated overnight at 4°C. The following day, the blocking solution with antibodies was removed and cells were washed three times with PBS. Secondary antibodies were diluted and applied in the same blocking solution for 1 h before the solution was removed. Samples were washed again with PBS, transferred to coverslips, and mounted with ProLong Gold Antifade with 4′,6-diamidino-2-phenylindole (DAPI; Life Technologies). Primary antibodies used include rabbit monoclonal anti-sarcomeric alpha actinin (Abcam, ab68167, Lot GR239387-29), mouse anti-N-Cadherin (BD Biosciences, 610921, Lot 7208869), mouse monocloncal anti-Na+-Ca2+-exchanger (NCX; Swant, R3F1), rabbit polyclonal anti-ventricular myosin light chain 2 (MLC2v; Proteintech, 10906-1-AP, Lot 00050697), and rabbit polycloncal anti-beta-1 adrenergic receptor (β1-AR; abcam, ab3442, Lot GR228889-7). Secondary antibodies include Alexa Fluor 488 (IgG1) goat anti-rabbit, Alexa Fluor 640 (IgG1) goat anti-rabbit, Alexa Flour 488 (IgG1) goat anti-mouse Alexa Flour 640 (IgG1) goat anti-mouse (Thermo Fisher Scientific). Immunolabeled samples were imaged using a Nikon A1RSi Confocal Microscope with an attached Photometrics CoolSNAP HQ2 camera. To capture representative images of the entire pattern, images of areas containing only lanes as well as areas containing lane and bridge regions were collected for all samples.

Corrected total cell fluorescence (CTCF) was used as the quantitative metric of fluorescence in Supplementary Figure S7C–E, first reported in McCloy, et al., and subsequently applied by ourselves and others (McCloy et al., 2014; Jakic et al., 2017; Brophy et al., 2022; Josvai et al., 2024). Composite images were divided into separate wavelength channels for analysis and CTCF was calculated: CTCF = (integrated density) – (area of selected cells × mean background fluorescence). The integrated density was calculated only over regions which contain cells to remove bias associated with the micropatterned geometry. A manual ROI was drawn to discriminate between cells and the background for calculations. An automated ImageJ macro was applied to split wavelength channels, measure the integrated density of fluorescence within the ROI, and calculate the mean background fluorescence. The results were calculated and normalized to CM Only samples in Excel and plotted using MATLAB.

### 2.11 Sarcomere alignment and cell morphology analysis

The open source scanning gradient Fourier transform (SGFT) method was used to quantify the myofibrillar alignment and sarcomere length of samples (Salick et al., 2020). Following acquisition, high-resolution images stained for sarcomeric alpha actinin were used to provide unbiased quantification of sarcomere organization. The following parameters were used as SGFT inputs (as determined in previous work) on ×100 objective images with a resolution of 4,096 × 4,096 pixels: 0.03 µm/pixel, a pattern size of 1.5, and a scan resolution of 16 (Salick et al., 2020; Stempien et al., 2024). Summary statistics were extracted from the software and plotted in MATLAB. For each stained sample, 4 locations were imaged and analyzed.

To perform cell size analysis, confocal image stacks of samples labeled for the membrane protein N-Cadherin, the sarcomere protein alpha actinin, and DAPI were binarized and a custom MATLAB algorithm was used to fill any gaps in N-Cadherin expression. For each image in a stack (which originate from different imaging z-heights of the same sample), the MATLAB function regionprops was used to define the cross sectional area (CSA) and centroid of each cell above a threshold. Because the step size between each image in a stack remained constant at 0.25 µm, centroids belonging to the same cell were mapped to one another from each image and multiplied by the step size for each image in a stack to calculate individual cell volumes (Supplementary Figure S2A). Regions in CM-CF samples which contained DAPI-labeled nuclei but lacked alpha actinin expression were identified as hiPSC-CFs, which do not contain contractile or sarcomeric proteins when healthy (Porter and Turner, 2009; Souders et al., 2009). These nuclei were identified abundantly in only the CM-CF coculture samples (implying a near pure population of hiPSC-CMs in CM Only and CF Conditioned treatments) and excluded from cell size calculations. This data was used in addition to estimate the final hiPSC-CM to hiPSC-CF ratio of 6.71:1 on day 18 of CM-CF coculture (87.2% included, 12.8% excluded; Supplementary Figure S2B). The 0.25 µm step size was also used as the resolution limit for tissue thickness measurements, which were taken from the z-stack thickness of confocal images.

### 2.12 Polymerase chain reaction (PCR)

Multiple samples of CM Only, CF Conditioned, and CM-CF Coculture were pooled and cryopreserved for PCR. Total RNA was isolated using a RNeasy Mini Kit with QIAshredder (Qiagen) and an on-column DNA digest with Turbo Dnase (ThermoFisher). 150 ng of purified RNA (all A260/A280 ≥2.0) was reverse transcribed using High-Capacity cDNA Kit (ThermoFisher). 7.5 ng of cDNA (and no RT control) for each condition was amplified by Phusion Polymerase (New England Biolabs) using Origene primer sequences for GAPDH (Origene HP205798), MYH7 (Origene HP200239) and TNNI3 (Origene HP200343), which are designed across exon-exon junctions to avoid genomic amplification. Primer sequences and expected amplification sizes are shown in Supplementary Table S1. PCR products were run on a 2% agarose gel and imaged.

### 2.13 Statistical methods

For brightfield microscopy, the reported sample size refers to biological replicates, rather than repeated acquisition at one timepoint within an individual micropatterned culture. For fluorescent microscopy, the number of locations imaged per micropattern sample is noted in the respective results section. Sample sizes and p values are reported in each figure legend. Data visualization and statistical analyses were performed using MATLAB. Directional statistics (circular variance and weighted anisotropy analysis) were performed using a modified version of the open-source MATLAB package CircStat v.1.21.0.0 (Berens, 2009). For statistical analysis of two groups, an unpaired two sample t-test was used. For analysis with more than two conditions a one-way ANOVA test was performed followed by multiple comparison test to perform pairwise comparisons between pairs of data using Tukey’s honestly significant difference criterion. Distributions were normal prior to statistical testing as determined by a Kolmogorov-Smirnov test for each data set (p < 0.05). Statistical significance was defined as *p < 0.05, **p <0 .01, ***p < 0.001.

## 3 Results

### 3.1 CM-CF culture and morphology

In this work, hiPSCs were differentiated into separate populations of hiPSC-CMs and hiPSC-CFs. hiPSC-CMs were seeded either alone or in coculture with hiPSC-CFs on platforms of micropatterned ECM. CM monoculture samples were split into two experimental groups: (1) CM Only in basal EB2 medium (described in methods) or (2) CF Conditioned samples in EB2 medium that had been conditioned by a CF monoculture for 48 h and then transferred to the patterned CMs. (A third CM monoculture subgroup, 0.5 CF Conditioned, was fed with a 1:1 dilution of hiPSC-CF conditioned medium and basal EB2, results included in supplemental.) The final experimental group, CM-CF Coculture, consisted of both CMs and CFs cultured on the micropatterned platform supplied with only basal EB2 medium for the duration of the experimental period (Figure 1). hiPSC-CMs and hiPSC-CFs were seeded at a ratio of 10:1 CM:CF on experimental day 0, with a final ratio of 6.89:1 CM:CF (Supplementary Figure S2B).

CMs cultured alone, including CM Only and CF Conditioned, remained within the defined boundaries of the ECM pattern through 18 days. CM-CF Coculture demonstrated progressive remodeling of the patterned ECM and migration to previously unoccupied regions of the substrate, resulting in an appearance similar to a confluent monolayer under brightfield microscopy (Figures 2A,B). Day 18 CM Only and CF Conditioned samples, lacking CFs in culture, demonstrate little alteration to the ECM topography. However, day 18 CM-CF Coculture demonstrates augmentation to the patterned matrix, including fibrillar ECM proteins deposited and remodeled by the CFs in culture (Figure 2C). As a result, by day 18 of the experimental period, CM-CF Coculture samples occupied 83.1% of the substrate, while CM Only and CF Conditioned samples occupied 65.8% and 67.9% of the substrate, respectively. 0.5 CF Conditioned samples (in a 1:1 dilution of CF conditioned medium and basal medium) also display no remodeling (Supplementary Figure S4A). When cultured on the micropatterned platform, CFs deposit fibrillar ECM to promote remodeling of the *in vitro* tissue as observed in this work and previously (Napiwocki et al., 2021b; Stempien et al., 2024). However, immunolabeled images reveal that the underlying CMs retain the organization afforded by the original pattern. Open-source SGFT analysis was used to provide unbiased quantification of sarcomere alignment across samples. No statistically significant differences existed in sarcomere organization between groups. Additionally, the sarcomere length was unaltered across conditions, with a median length between 1.71 and 1.75 µm (Figure 2D), near the value reported in healthy native cardiac muscle (Ter Keurs et al., 1980; Crocini and Gotthardt, 2021). Other cardiac tissue models designed to enhance maturation and promote sarcomere elongation, such as 2D PDMS and ECT systems, found comparable results with sarcomere lengths ranging from 1.67 to 1.82 µm (Siddique et al., 2022; Strimaityte et al., 2022; Wang et al., 2023; Rossler et al., 2024). N-cadherin-stained images were used to define the boundaries of individual cells and DAPI was used to identify nuclei. There were no changes in the maximum cross-sectional area of cells or nuclei, the tissue thickness, or the cellular volume across groups (Figure 2E). These data demonstrate that despite varying experimental conditions and the ECM remodeling in CM-CF Coculture samples, the structural organization of the cells remains unaltered. While myofibrillar organization does influence the mechanical capabilities of the native myocardium, its role in the contractile amplitude or kinetics in the *in vitro* system remains unclear.

### 3.2 CM contractile behavior improves with CF coculture

**FIGURE 3:**
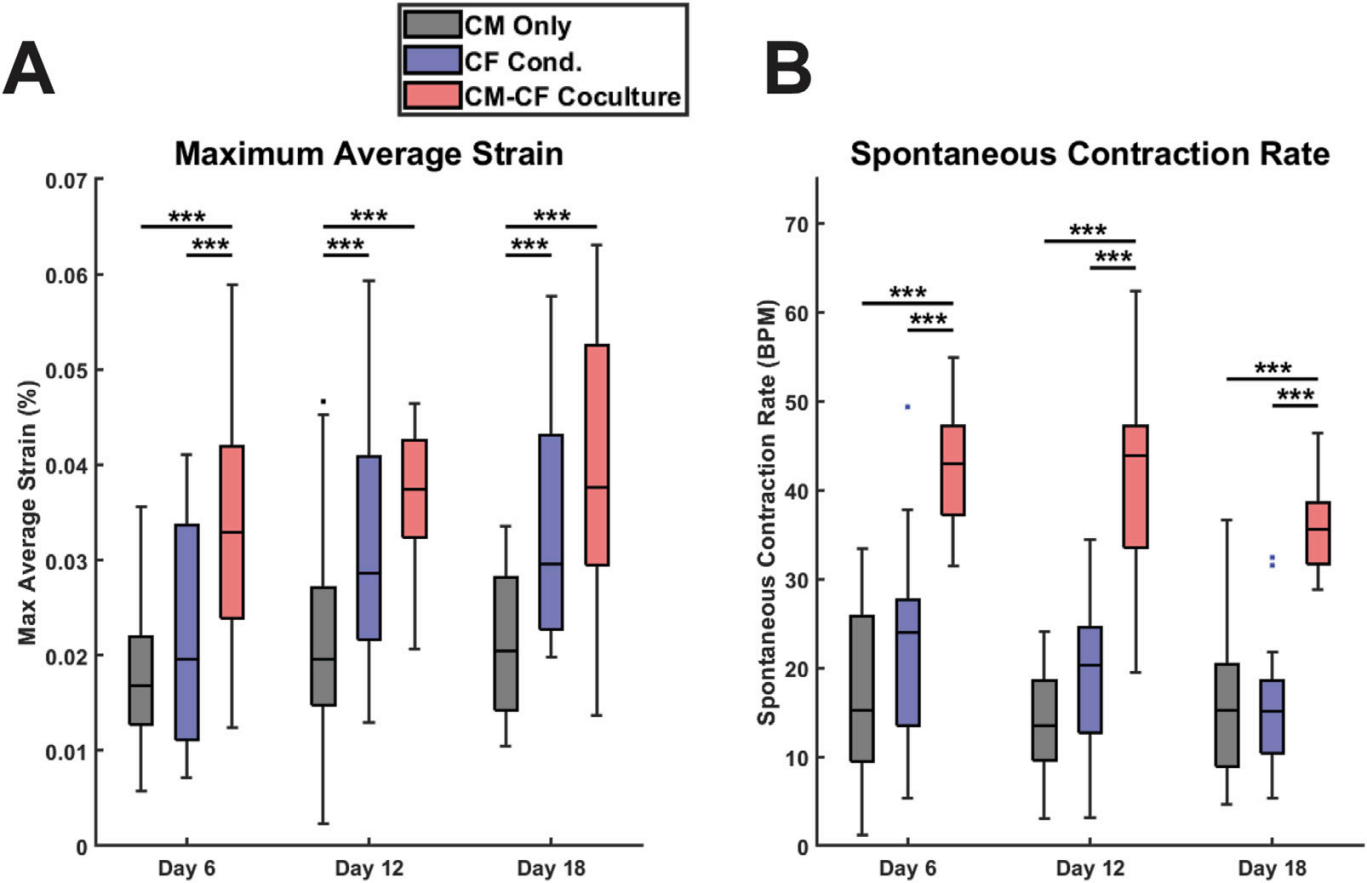
CF Conditioned medium and CM-CF Coculture exert differential effects on contractile behavior. **(A)** The maximum contractile strain achieved by each group on days 6, 12, and 18. The maximum contractile strain is the peak of the second principal strain averaged across the full field of imaging as calculated by DIC. **(B)** The spontaneous rate of contraction for each condition at each experimental timepoint. N = CM Only: d6 = 47, d12 = 43, d18 = 28; CF Conditioned: d6 = 38, d12 = 35, d18 = 23; CM-CF Coculture: d6 = 20, d12 = 23, d18 = 17. A minimum of five unique hiPSC-CM differentiations were used for each experimental group.

Videos of spontaneous coordinated cell contractions were captured using brightfield microscopy, and the contractile strains were calculated using digital image correlation (DIC) and averaged across the full field of acquisition, excluding regions that were not occupied by cells. All contractile data included is spontaneous, no electrical pacing was used in this work. This dataset also includes a thorough retrospective analysis of the contractile behavior of CM Only and CM-CF Coculture samples from our previous work in Stempien et al., 2024. These datasets were previously analyzed solely for contraction magnitude; however, in the present study, we expand the analysis to include both contractile alignment and kinetics. Beginning on day 6 and at each time point thereafter, there was a significant increase in the peak strain generated in the CM-CF Coculture compared to the CM Only, with CM-CF Coculture increased by 96%, 91%, and 83% on days 6, 12, and 18 (Figure 3A). However, a significant difference between the CF Conditioned and CM Only was present only at the day 12 and day 18 timepoints, and the magnitude of the difference was less pronounced (46% and 45% on days 12 and 18, respectively). On day 6, the peak strain was increased in the CM-CF Coculture compared to the CF Conditioned. CM-CF Coculture resulted in an increased rate of contraction at each experimental timepoint while CF Conditioned medium had no effect on rate (Figure 3B). No correlation was observed between the amplitude of contractile strain and the spontaneous rate of contraction (Supplementary Figure S5). Interestingly, the difference in contractile strain between CM Only and the diluted 0.5 CF Conditioned group only reached significance on day 18, suggesting that a temporal dosage response may exist (Supplementary Figure S4B).

### 3.3 Displacement trajectory alignment enhanced in CM-CF

**FIGURE 4:**
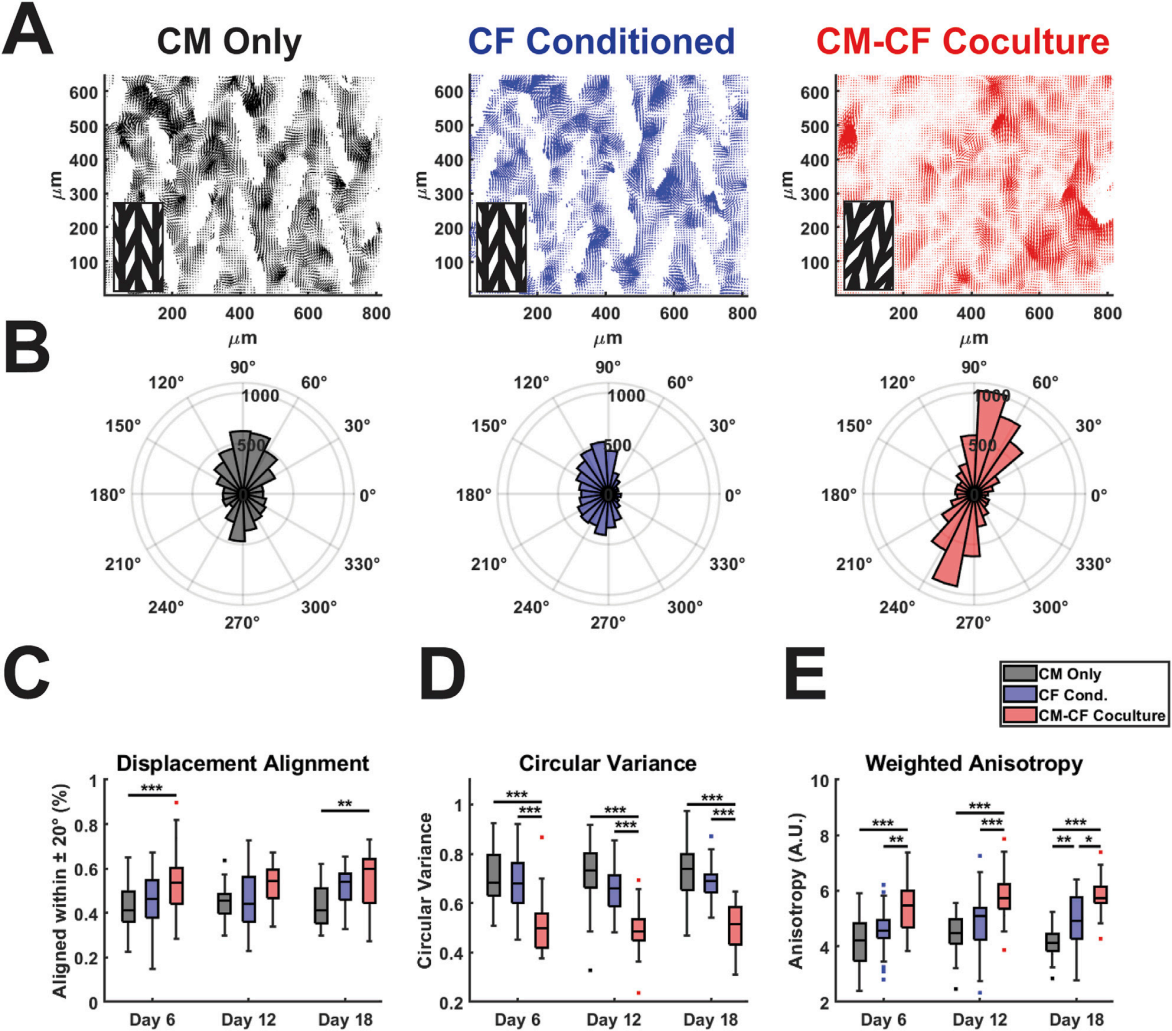
CM-CF Coculture promotes displacement trajectory alignment. **(A)** Representative displacement vector maps for CM Only (left), CF Conditioned (center) and CM-CF Coculture (right). Inset denotes the orientation of the original micropattern design. Vector maps correspond to the day 18 samples in Figure 2A. **(B)** Representative polar histograms (corresponding to the vector maps in Figure 4A) for each experimental group, demonstrating the alignment of displacement trajectories. Polar histograms account only for displacement trajectory and not magnitude, though displacements with a magnitude less than 75% of the median for an individual sample were excluded from summary measurements in Figures 4C–E. **(C)** Displacement trajectory alignment (defined as the percentage of displacements aligned within 20 degrees of the most predominant principal strain orientation), **(D)** circular variance of displacement angles (ranges from 0 to 1), and **(E)** weighted anisotropy (defined as the sum of all vector magnitudes aligned within 20 degrees of the predominant orientation divided by the sum of all unaligned vector magnitudes). N = CM Only: d6 = 47, d12 = 43, d18 = 28; CF Conditioned: d6 = 38, d12 = 35, d18 = 23; CM-CF Coculture: d6 = 20, d12 = 23, d18 = 17. A minimum of 5 unique hiPSC-CM differentiations were used for each experimental group.

Native cardiac muscle exhibits anisotropic organization, resulting in a preferential orientation of electrical signal propagation and contraction behavior (Kotadia et al., 2020; Veldhuizen et al., 2020; Dwyer and Coulombe, 2021). To understand other features of contractile behavior, we investigated the organization and alignment of the displacements identified by DIC. We have previously demonstrated that the micropatterned culture system used in this work results in anisotropic electrical conduction and organization in CM monocultures (Napiwocki et al., 2021b; Stempien et al., 2024). To extend the utility of DIC analysis and explore the effects of CM-CF Coculture and CF Conditioned medium on contractile anisotropy, displacement trajectory analysis was performed. For all experimental groups, displacement trajectories tended to align in the primary orientation of the original micropatterned design (Figures 4A,B, representative vector maps and rose plots correspond to the day 18 samples in Figure 2A). CM-CF Coculture had significantly increased displacement trajectory alignment, defined as the percentage of displacement vector trajectories aligned within 20 degrees of the most predominant principal strain orientation, in comparison to CM Only control on days 6 and 18 (Figure 4C). There were no such differences between CF Conditioned and CM Only groups at any timepoint. The circular variance of displacement trajectories angles was also used as a metric of contractile alignment, with CM-CF Coculture having a significantly reduced variance in comparison to both CM Only and CF Conditioned at all time points (Figure 4D). All three experimental groups demonstrated significant contractile displacement anisotropy, with median weighted anisotropy values greater than 4 at all time points (defined as the sum of all vector magnitudes aligned within 20 degrees of the most predominant principal strain orientation divided by the sum of all unaligned vector magnitudes). However, the degree of anisotropy was increased in CM-CF Coculture compared to CM Only and CF Conditioned at all experimental timepoints (Figure 4E). There was no difference in anisotropy between CM Only and CF Conditioned on days 6 and 12, though a significant increase was present by day 18. Taken together, these data indicate that CM-CF coculture promotes overall contractile organization (increased alignment, decreased variance, and increased anisotropy) significantly more than CF Conditioned in comparison to a CM Only control and that CF Conditioned medium may only promote such anisotropic alignment at late time points.

### 3.4 Coculture alters CM contractile kinetics

**FIGURE 5:**
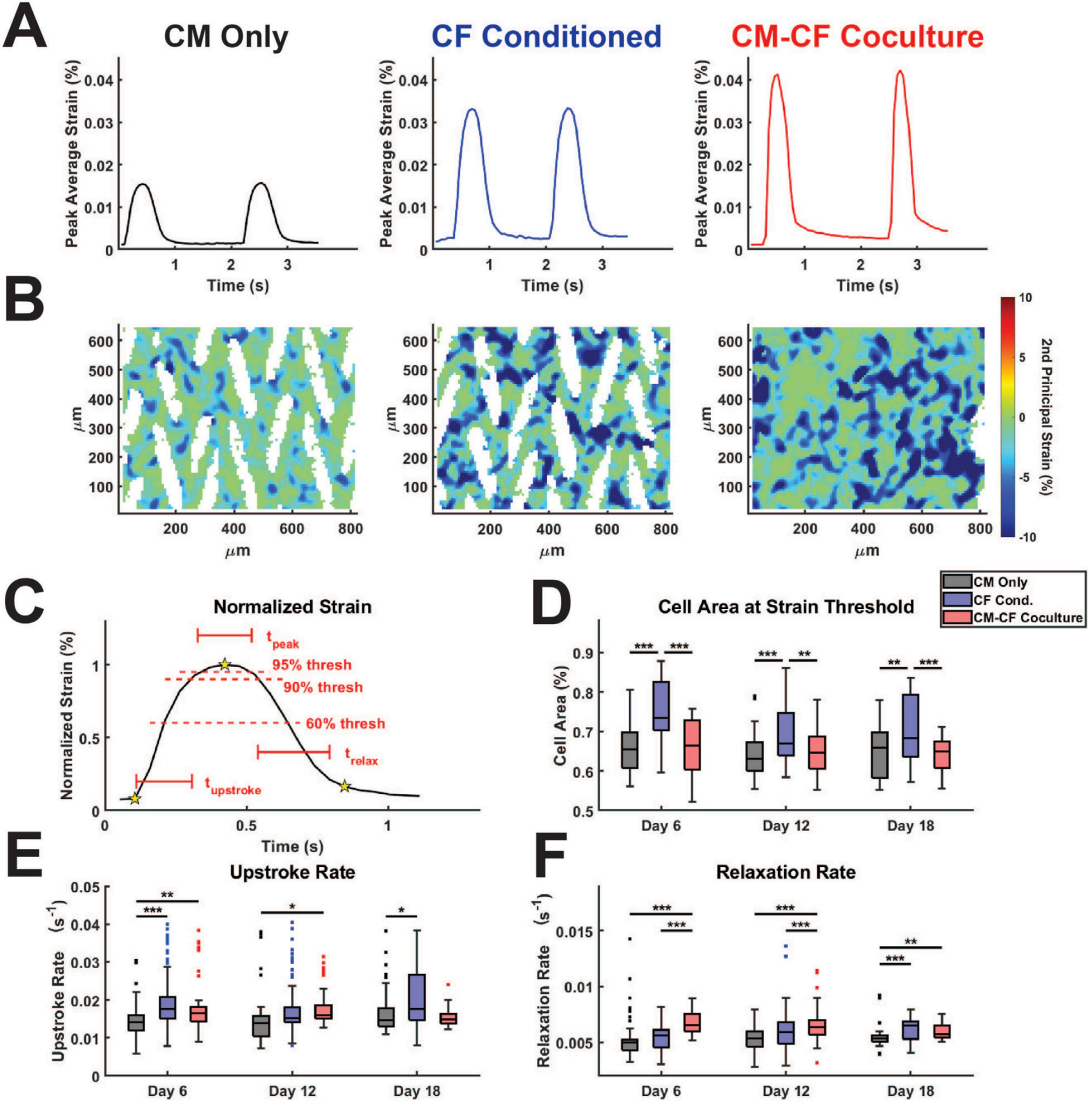
CF Conditioned Medium and CM-CF Coculture alter the contractile and relaxation kinetics of hiPSC-CMs. **(A)** Representative strain traces and **(B)** strain maps, corresponding to the day 18 samples in Figures 2A, 4A. Representative strain maps are from the peak of the first contraction shown in Figure 5A. **(C)** Normalized strain schematic demonstrating initiation (first start), peak (second star) and rest (third star) points used for kinetic calculations and to determine the 60% and 90% threshold values. **(D)** Cell area reaching the 60% strain threshold at the maximum strain state, used as a metric to approximate the percentage of cells achieving considerable contraction states. **(E)** Contraction upstroke strain rate and **(F)** relaxation strain rate. N = CM Only: d6 = 47, d12 = 43, d18 = 28; CF Conditioned: d6 = 38, d12 = 35, d18 = 23; CM-CF Coculture: d6 = 20, d12 = 23, d18 = 17. A minimum of 5 unique hiPSC-CM differentiations were used for each experimental group.

We have previously demonstrated that CM-CF Coculture results in a significantly increased amplitude of contractile strain in comparison to CM monocultures (Stempien et al., 2024; Napiwocki et al., 2021b). Yet, analogous to cardiac Ca^2+^ handling, the time-dependent kinetics of contraction behavior may be considered equally important as the absolute amplitude of contraction behavior (Janssen, 2010; Kijlstra et al., 2015). To perform such analyses, we used custom-built software for this work, as described in the methods, to probe the kinetics and heterogeneity of the contractile behavior identified through DIC. Representative traces of CM Only, CF Conditioned, and CM-CF Coculture strain behavior allow visualization of both the changes to strain amplitude as well as the differential kinetics between treatment groups (Figure 5A). Additionally, representative heatmaps of strain at the maximum contraction amplitude may be used to visualize strain behavior. While the full-field averaged strain displays clear contraction and relaxation behavior, much heterogeneity exists in the regional magnitudes of strain, with some regions remaining at a near zero principal strain state (Figure 5B, corresponding to the starred frame in Figure 5A). The peak of strain, as well as the initiation and relaxation points, may be used to explore the kinetics of spontaneous contractile behavior (Figure 5C). The cell area reaching the 60% normalized strain threshold is used as a metric for the relative homogeneity of contraction capabilities across the field of imaging. Masked regions (white areas which contain no cells) are excluded from such calculations.

CF Conditioned samples had a significantly higher area reaching threshold in comparison to CM Only control and CM-CF Coculture, though we hypothesize that this increase may occur through different mechanisms (Figure 5D). The area reaching threshold in CF Conditioned may be increased in comparison to CM Only due to the increased mechanical capabilities of these cells, as demonstrated in the absolute strain measurements. In contrast, the increase in CF Conditioned over CM-CF Coculture is likely a result of the mechanically inactive CFs present in the coculture group. CFs occupy the gaps from the original micropattern but do not actively contribute to the contractile strain. As a result, the percentage of area reaching threshold in CM-CF Coculture samples is decreased in comparison to CF Conditioned, which contains no fibroblasts in culture. There were no differences in the time spent at peak (time greater than the 90% strain threshold) across any treatments or time points, though differences may not be distinguishable due to the ∼50 ms resolution of the imaging system (Supplementary Figure S6A). The upstroke rate, the normalized slope from initiation to the peak of contraction, was significantly increased in CF Conditioned compared to CM Only on days 6 and 18, and in CM-CF Coculture compared to CM Only on days 6 and 12 (Figure 5E). CM-CF Coculture also displayed improved relaxation kinetics, with significantly shorter relaxation times and increased relaxation rates on days 6 and 12 in comparison to CM Only and CF Conditioned (Figure 5F; Supplementary Figure S6B). The relaxation time was significantly faster in CM-CF Coculture compared to CM Only on day 18, but no difference existed between CM-CF Coculture and CF Conditioned. Similarly, both CM-CF Coculture and CF Conditioned had increased relaxation rates on day 18 compared to CM Only control. These data emphasize that both coculture and conditioned medium improve the contractile capabilities of hiPSC-CMs, while CM-CF Coculture seems to preferentially influence the relaxation properties of the *in vitro* samples, especially at earlier timepoints.

### 3.5 Conditioned medium effects remain after FCM removal

**FIGURE 6:**
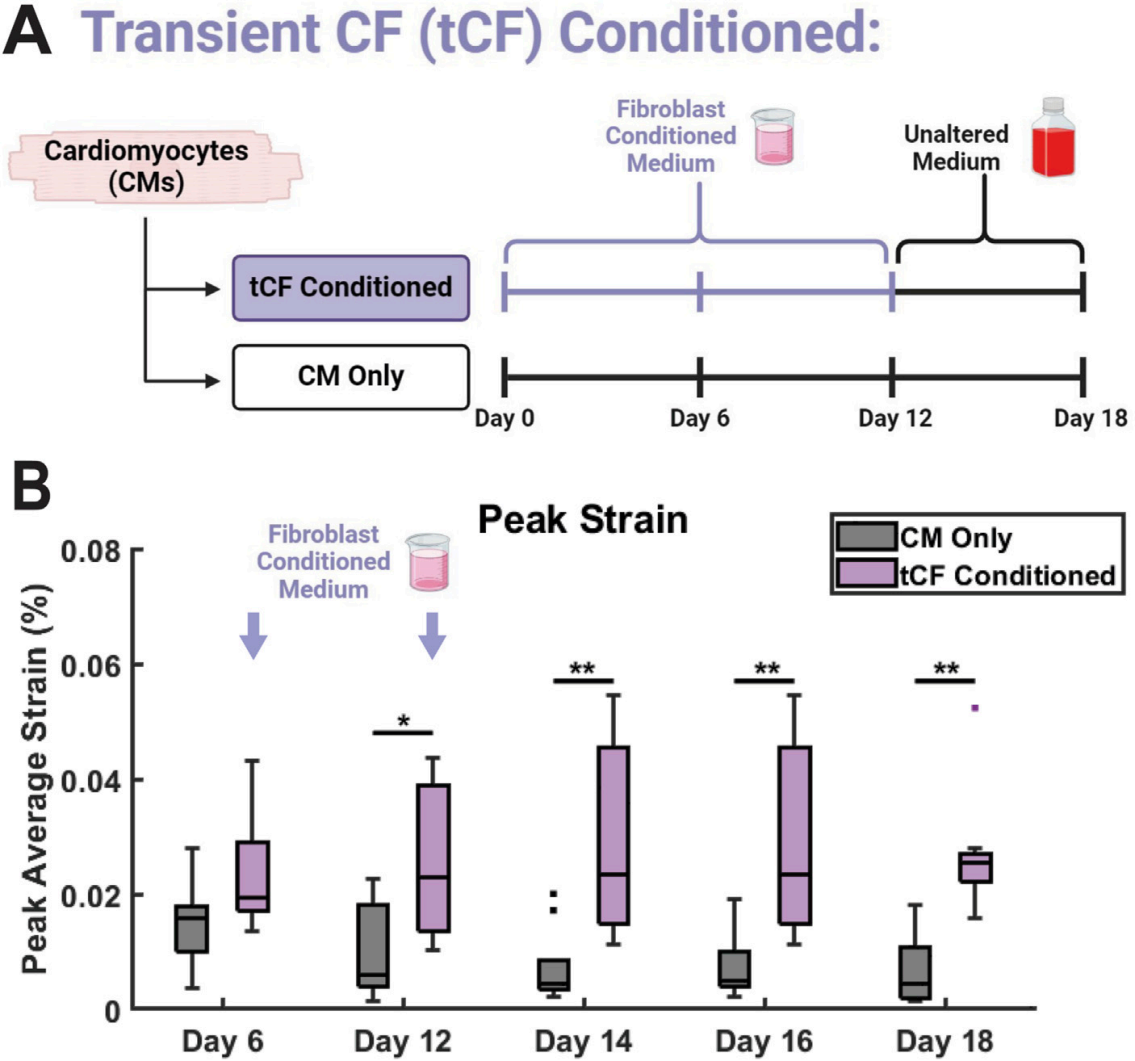
Transient CF (tCF) Conditioned medium experimental results. **(A)** Experimental design for tCF Conditioned. Samples are provided with fibroblast conditioned medium from days 0 to 10, then provided with only basal medium until day 18. CM Only samples were provided with only basal medium from days 0 through 18. **(B)** The maximum contractile strain achieved on days 6, 12, 14, 16, and 18 as calculated by DIC. N = 10 for both groups at all time points. A minimum of 3 unique hiPSC-CM differentiations were used for each experimental group.

A hallmark of CM maturation is increased force generation and contractile capability (Ahmed et al., 2020). Coculture between hiPSC-CMs and non-cardiomyocyte cells, including CFs, has previously been shown to aid in CM maturation (Yoshida et al., 2018; Abecasis et al., 2019; Giacomelli et al., 2020). In our model, if the observed differences in contractile strain in the CF Conditioned group are the result of functional maturation, such effects would remain after the replacement of FCM with basal medium. Conversely, if the enhancements to CM mechanical output are the result of transient signals through adrenergic stimulation or another CF-mediated mechanism, the effects would be lost after FCM was replaced with basal media. To probe these effects, we provided CM monocultures with CF Conditioned medium in the same manner as previously described from days 0–10. Subsequently, from days 12–18 (no medium change on day 11), these samples were provided with basal, unconditioned, medium in the same manner as the CM Only control. As a result, these samples are referred to as transient CF (tCF) Conditioned (Figure 6A). As observed previously, there was no change in peak strain between CM Only and tCF Conditioned on day 6, with the first statistical difference manifesting by experimental day 12 (Figure 6B). On days 14, 16, and 18 (after the samples had been supplied with only basal, unconditioned medium since day 12), the increase in peak strain in the tCF Conditioned remained significant. Together, this supports the hypothesis that CF Conditioned medium induces a sustained functional enhancement in hiPSC-CMs, resulting in increased force generation and contractile capability. Additionally, this indicates that the strain effects observed in the CF Conditioned and CM-CF Coculture have the potential to be the result of CM functional maturation. Supporting this hypothesis, we observed the expression of the adult isoform genes *MYH7* and *TNNI3* in all micropatterned groups (Supplementary Figure S7). Further, fluorescence-based analysis revealed elevated expression levels of key maturation markers in the CM-CF Coculture group relative to CM Only controls, including ventricular myosin light chain 2 (MLC2v), the Na^+^/Ca^2+^ exchanger (NCX), and the β1-adrenergic receptor (β1-AR), each of which is associated with enhanced iPSC-CM maturation (Supplementary Figure S8).

## 4 Discussion

While CMs are the contractile cells of the heart, CFs contribute to the structural, biochemical, mechanical, and electrical properties of the myocardium (Camelliti et al., 2005; Sadoshima and Weiss, 2014; Sun et al., 2023; Harrington and Moore-Morris, 2024). We sought to characterize the effects of CM-CF Coculture and CF Conditioned medium on the mechanical function of hiPSC-CMs seeded on a patterned substrate and to determine if observed alterations were the result of paracrine soluble signalling or direct cell-to-cell effects. Only CM-CF Coculture samples exhibited laminin reorganization, appearing similar to a monolayer due to the ability of CFs to deposit and remodel the substrate ECM (Napiwocki et al., 2021b; Bekedam et al., 2024). However, the structural properties of CMs, including sarcomere alignment and length, cell and nuclei area, and tissue thickness were unaltered across experimental groups. Together, this indicates that alterations to the mechanical capabilities of micropatterned hiPSC-CMs may result from changes to cellular physiology including metabolic or transcriptional alterations reported by others, as no changes were observed to the structural or organizational characteristics (Fu et al., 2011; Lundy et al., 2013; Feyen et al., 2020).

We next demonstrated that CM-CF Coculture increases the maximum contractile strain and spontaneous rate of CMs at all time points. While these observations match the literature and our previous findings, a mechanism underlying this phenomenon has yet to be identified and may relate to differential CM ion channel expression induced by CM-CF Coculture (Zhang et al., 2013; Kim et al., 2015; Giacomelli et al., 2020). CF Conditioned medium has no effect on spontaneous rate and only results in increased maximum strain by days 12 and 18, while treatment with a 1:1 dilution of CF Conditioned and basal media delayed the increase in peak strain until day 18, suggesting the effects of FCM are both time- and dosage-dependent. Additionally, while the focus of this study was specifically on the spontaneous electrical activity of iPSC-CMs, we have also previously demonstrated the capability to achieve full-field coordinated contraction with electrical pacing of the system (Napiwocki et al., 2021a; De Lange et al., 2023).

In native, healthy tissue, myocardial organization and contraction display uniform and anisotropic organization, thus *in vitro* and *in silico* models of the myocardium must strive to recapitulate such characteristics (Caggiano et al., 2018; Avazmohammadi et al., 2019; Jorba et al., 2021). To further explore CF-mediated effects on the dynamics of contractile behavior, we performed displacement trajectory and contractile kinetics analyses. CM-CF Coculture exhibited more homogenous contractions, with increased alignment and decreased circular variance of the displacement vectors in comparison to CF Conditioned and CM Only control. While all patterned samples demonstrated anisotropy of contraction, CM-CF Coculture had a significantly higher weighted anisotropy compared to both other treatments at all time points. The anisotropy of CF Conditioned was only increased on day 18 in comparison to CM Only, demonstrating the Coculture preferentially promotes contractile alignment. We have previously shown that hiPSC-CFs cultured on this platform deposit unidirectional and fibrillar ECM proteins, including fibronectin and collagen 1A (Napiwocki et al., 2021a; Stempien et al., 2024), while others have reported ECM fiber alignment to influence hiPSC-CM maturation and organization (Nakayama et al., 2014; Han et al., 2016; Batalov et al., 2021) Thus, we hypothesize that the increased contractile alignment in CM-CF coculture may be related to the deposition of fibrillar ECM by hiPSC-CFs which provides enhanced structural cues to CMs.

Electrophysiologists have long been interested in the temporal kinetics of cardiac action potentials and Ca^2+^ handling and the role these dynamics play in the function of the heart (recently, Ernst et al., 2022; Lee et al., 2023; Clark et al., 2024). In this work, we applied a similar algorithm to the techniques used in electrophysiological quantification to explore the heterogeneity and kinetics of strain behavior. Each treatment displays heterogeneous amplitudes of contractile strain, as visualized in the representative heatmaps. CF Conditioned samples had the greatest percentage of cell areas reaching the 60% strain threshold, and we posit that the difference between CM Only and CF Conditioned is the result of the increased mechanical capabilities of conditioned CMs, manifesting as a larger percentage of cells achieving threshold contractions. In addition, the difference between CF Conditioned and CM-CF Coculture results from the presence of CFs in coculture, which are not mechanically active and will only achieve subthreshold strains due to the contraction of neighboring cells. Both CF Conditioned and CM-CF Coculture resulted in a faster strain upstroke rate at multiple timepoints, suggesting that paracrine signalling may be sufficient to improve the kinetics of active contraction. Only CM-CF Coculture altered the relaxation time and rate, while CF Conditioned had no effect at early timepoints. These data indicate that the presence of CFs in culture promotes both the active cellular contractile properties and the passive strain relaxation kinetics. We have demonstrated that CFs cultured on the micropatterned substrates deposit and remodel fibrillar ECM proteins (Stempien et al., 2024), suggesting that these fiber networks play a role as a passive mechanical restitution mechanism during relaxation, though further work is necessary to explore this phenomenon.

In the absence of structural alterations, other determinants of cardiac contractility must underlie the observed improvements in contractile function in the CM-CF Coculture and CF Conditioned groups (Penefsky, 1994; Wehrens and Marks, 2004; Lyasnikova et al., 2024; Romero et al., 2024). hiPSC-CM maturation is associated with several molecular shifts, including the transition of myosin isoforms (MYH6 to MYH7, MLC2a to MLC2v) and troponinI isoforms (TNNI1 to TNNI3) (Lee et al., 2011; Bedada et al., 2014; Tiburcy et al., 2017; Yoshida et al., 2018; Rapöhn et al., 2024). These transitions are accompanied by increased expression of ion channels and the β1-adrenergic receptor (β1-AR) (Yang et al., 2014; LaBarge et al., 2019; Hasan et al., 2020). While the specific MYH or TNI isoform may influence the mechanical capabilities of cardiomyocytes, these effects are difficult to decouple from other maturation processes that occur concurrently. However, we observed expression of *MYH7* and *TNNI3* in all micropatterned samples, as well as increased fluorescence-based expression of MLC2v, NCX, and β1-AR in CM-CF Coculture compared to CM Only. These findings are consistent with a more mature CM phenotype (Lundy et al., 2013; Miki et al., 2021). To further determine if the observed effects are the result of hiPSC-CM functional maturation through a CF-mediated mechanism, or a transient effect unrelated to CM maturation, we provided hiPSC-CM monocultures with CF Conditioned medium until day 12, when contractile differences first manifested, and subsequently only basal medium until day 18 (referred to as tCF Conditioned). From days 14 through 18, the changes in peak contractile strain remained with little difference in the amplitude of increase across time points. These data indicate that the effects of CF Conditioned medium (and therefore CM-CF Coculture) induce sustained functional enhancement and potential maturation that does not revert in response to the removal of CF soluble factors from the system.

Taken together, these results suggest that hiPSC-CFs influence hiPSC-CM mechanical activity through a mechanism that requires both paracrine signalling and direct cell-to-cell interactions, and that these effects push CM toward functional maturity. Our CF Conditioned findings indicate that soluble signalling is sufficient to enhance the amplitude and upstroke kinetics of contractile strain. In contrast, the presence of CFs and the resulting cell-to-cell or matrix interactions are necessary to increase CM spontaneous rate, as well as improve contraction displacement alignment, variance, anisotropy, and relaxation kinetics. Our group and others have previously demonstrated cardiomyocyte mechanical function as a metric for the identification of genetic disease phenotypes (in both primary cardiomyopathy and electrophysiological disorders) as well as to cardiotropic drugs (Dainis et al., 2020; Stempien et al., 2022; De Lange et al., 2023; Dou et al., 2023). Here, we present evidence that *in vitro* mechanical output may serve as a viable, non-invasive, and label-free proxy for hiPSC-CM functional maturity.

2D *in vitro* tissue models, such as the one described, exist with inherent limitations including simplified architecture and cell environment (Caddeo et al., 2017). The results of this work come from a single hiPSC line and should be further validated in additional iPSC lines. In addition, the engineered culture platform described here is fabricated with Matrigel, which is heterogenous and xenogenic (Kozlowski et al., 2021). Although the platform was designed as a tool for functional disease modelling and drug screening that enables relatively large-scale analysis and data acquisition for an *in vitro* system, further development would be required to increase throughput and add multiplexing capability. While the CM Only-CF Conditioned-CM-CF Coculture paradigm is useful to decouple some of the soluble and cell-to-cell effects, the two mechanisms are inextricably intertwined through the sequestration of growth factors and fibroblast communication and a simplified system may not fully capture this complexity (Teixeira et al., 2020; Trujillo et al., 2020). Finally, all conclusions are based on morphological or contractile results, and while we have identified an increase in contractile strain amplitude, we have not defined the change mechanistically beyond the role of paracrine signalling.

Our future work will explore the mechanisms of the differential enhancement through characterization of the FCM and the CM-CF Coculture microenvironment, as has been described previously (Moore and Emili, 2021; Nakamura et al., 2021). This mechanism may be related to protein isoform transitions, a metabolic shift from glycolysis to fatty acid oxidation, or another mechanism associated with CM maturation (Tohyama et al., 2013; Bedada et al., 2014; Yoshida et al., 2018; Sun et al., 2023). These findings, in tandem with our and others’ previous work, demonstrate the importance of multicellular, chamber-specific models of the human heart to probe both fundamental biology and pathophysiological mechanisms (Rizvi et al., 2016; Brown et al., 2024; Dewar et al., 2024). We also anticipate the use of the coculture model to investigate disease mechanisms, as the cellular constituents are each derived from hiPSCs that may be patient-specific (Tanaka et al., 2015; Reilly et al., 2022).

## Data Availability

The raw data supporting the conclusions of this article will be made available by the authors, without undue reservation.

## Abbreviations

hiPSC, human induced pluripotent stem cell; CM, cardiomyocyte; CF, cardiac fibroblast; ECM, extracellular matrix; CM-CF, cardiomyocyte-cardiac fibroblast coculture; FCM, cardiac fibroblast conditioned medium.
