# Supplementary material for: hiPSC-derived cardiac fibroblasts dynamically enhance the mechanical function of hiPSC-derived cardiomyocytes on an engineered substrate
Source: Front Bioeng Biotechnol. 2025 May 23;13:1546483. doi: 10.3389/fbioe.2025.1546483 (PMC12141862; doi:10.3389/fbioe.2025.1546483)
Supplement: Supplementary file 1 [file DataSheet1.docx]

Supplementary Material: Josvai et al, 2025

# Supplementary Figures

## Supplementary Figure 1


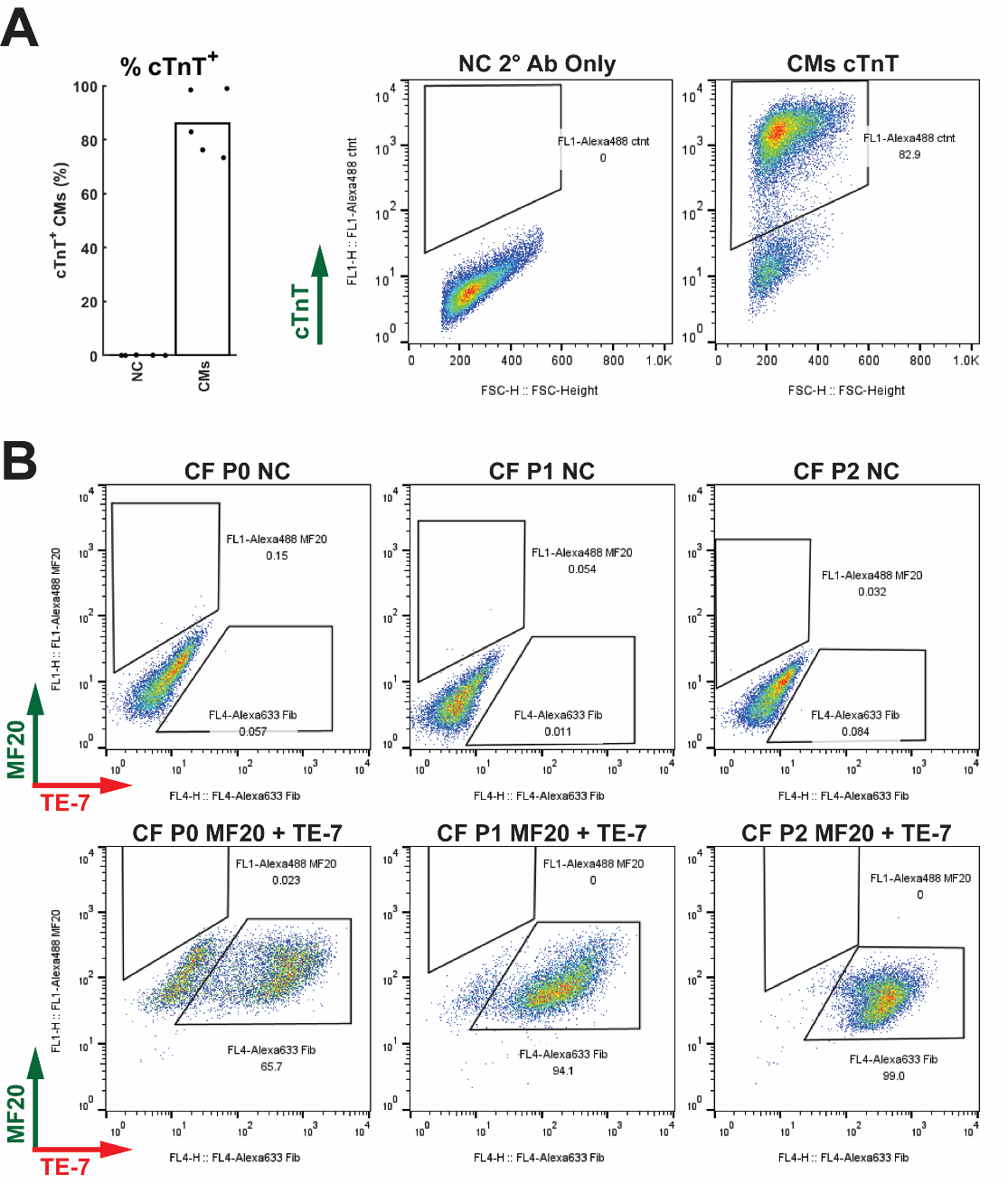


**Supplementary Figure 1:** Flow cytometry analysis of hiPSC-CM and hiPSC-CF differentiation. **(A)** hiPSC-CMs differentiated from hiPSCs between passage 37 and passage 70 and labeled for CM-specific cTnT showed an average cTnT^+^ of 85.9%. 2° antibody only negative control (NC) had a cTnT^+^ of <0.1% (left, N = 5). Representative NC (center) and cTnT (right) flow cytometry plots. **(B)** Representative flow cytometry plots of hiPSC-CFs NC (top) or labeled with CM-specific myosin heavy chain (MF20) or CF-specific TE-7. Day 20 CFs (P0, left column) are 65.7% TE-7^+^. After passage 2 (P2, right column), CFs are 99.0% TE-7^+^.

## Supplementary Figure 2


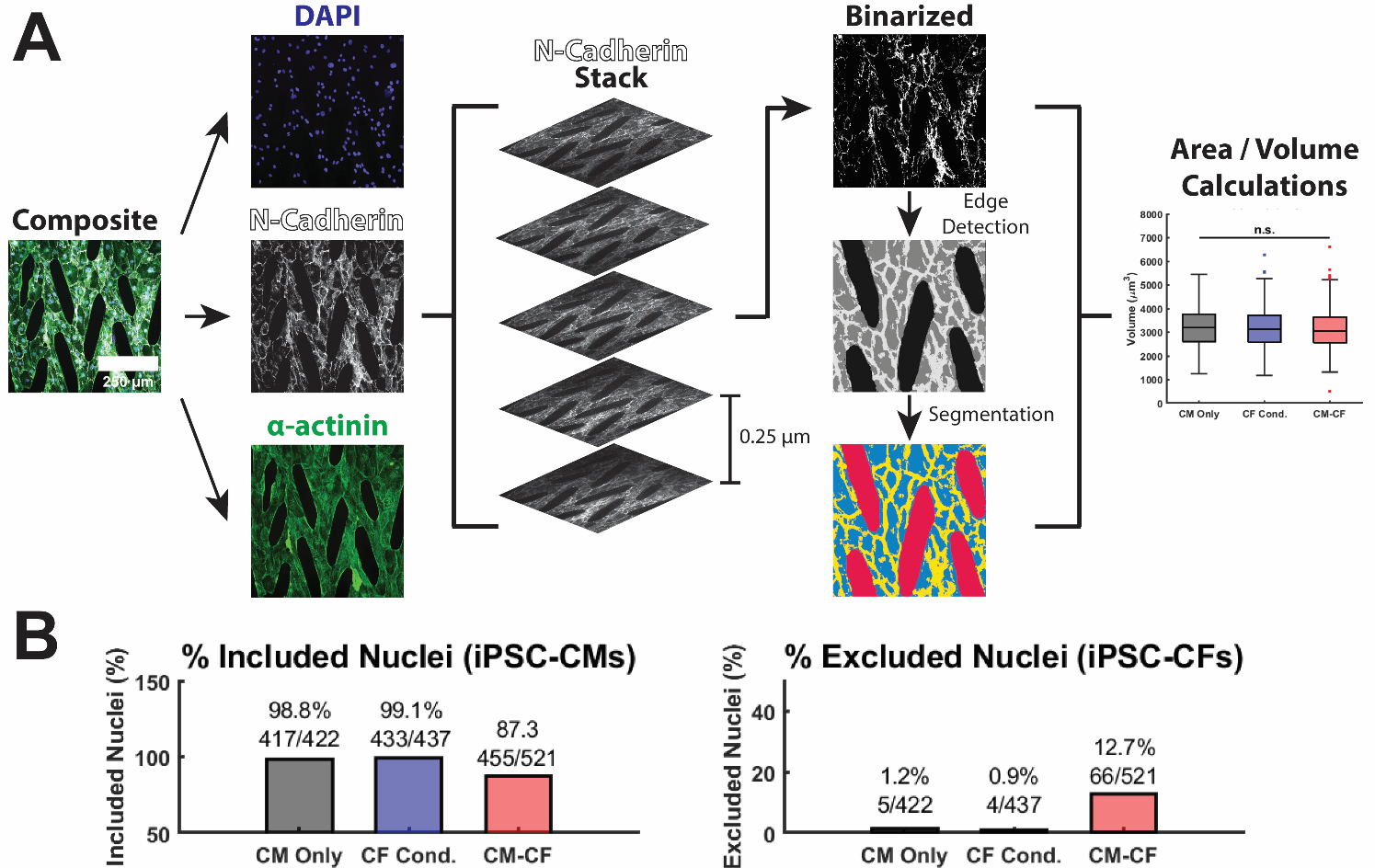


**Supplementary Figure 2.** Calculations of cell structure and morphology. **(A)** Image stacks of N-Cadherin-stained samples with a vertical step height of 0.25 µm are binarized, and edge detection is used to fill in remaining gaps in peripheral N-Cadherin staining. Unbiased segmentation is performed using MATLAB. Individual cell areas were multiplied by the step size of 0.25 µm and summed over the total z height of the sample image stack to calculate cell area. Only N-Cadherin staining is used for area and volume quantification. **(B)** Identification of day 18 hiPSC-CMs and hiPSC-CFs for cell structure calculations. Nuclei in regions expressing sarcomeric alpha actinin are included as CMs and used in calculations. Conversely, nuclei in regions which do not express alpha actinin are assumed to be hiPSC-CFs and are excluded from calculations. The relative ratios of CMs to non-CMs were 83.4:1 (CM Only), 108.3:1 (CF Conditioned), and 6.89:1 (CMCF Coculture).

## Supplementary Figure 3


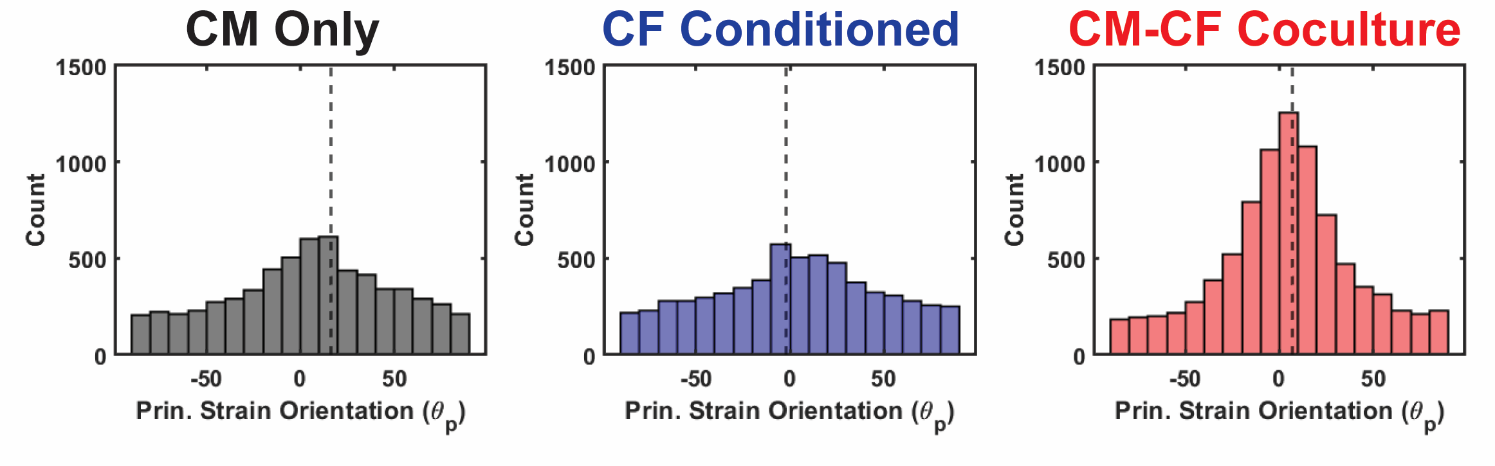


**Supplementary Figure 3.** Displacement trajectory alignment orientation selection. Representative histograms of the principal strain orientations identified by DIC software. Dotted vertical lines represent the predominant orientation used for displacement trajectory alignment calculations. Histograms correspond to the CM Only, CF Conditioned, and CM-CF Coculture samples shown in **Figure 2A, 4A, and 5A**.

## Supplementary Figure 4


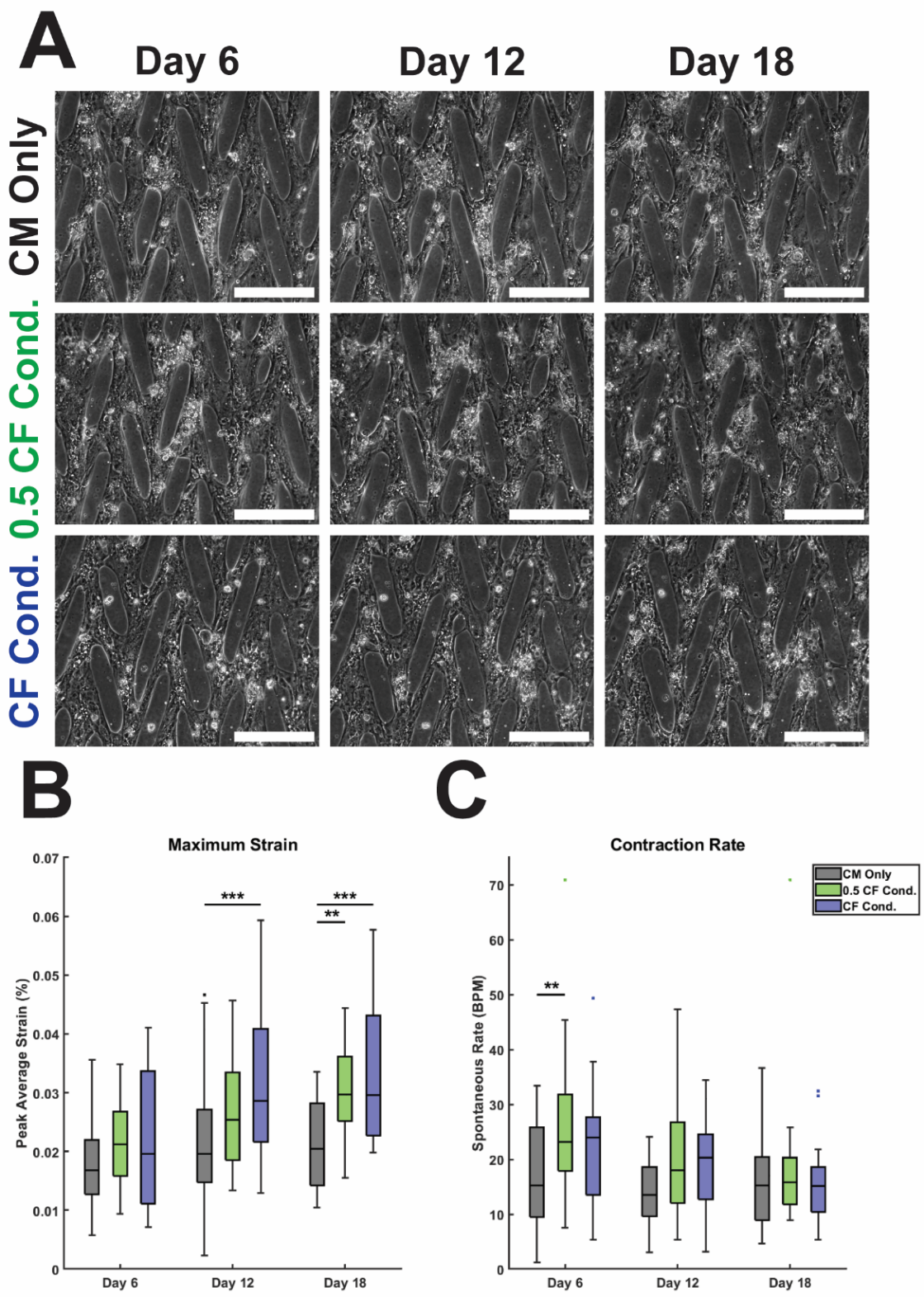


**Supplementary Figure 4.** Conditioned media response demonstrates dose dependence **(A)** Representative Day 6 (left), Day 12 (center column), and Day 18 (right) images of CM Only (top), 0.5 CF Conditioned (center row), and CF Conditioned (bottom) groups. Scale bars = 250 µm. **(B)** The maximum contractile strain achieved by each group on days 6, 12, and 18. **(C)** The spontaneous rate of contraction for each condition at each experimental timepoint. N = CM Only: d6 = 47, d12 = 43, d18 = 28; 0.5 CF Conditioned: d6 = 33, d12 = 29, d18 = 28; CF Conditioned: d6 = 38, d12 = 35, d18 = 23.

## Supplementary Figure 5

**
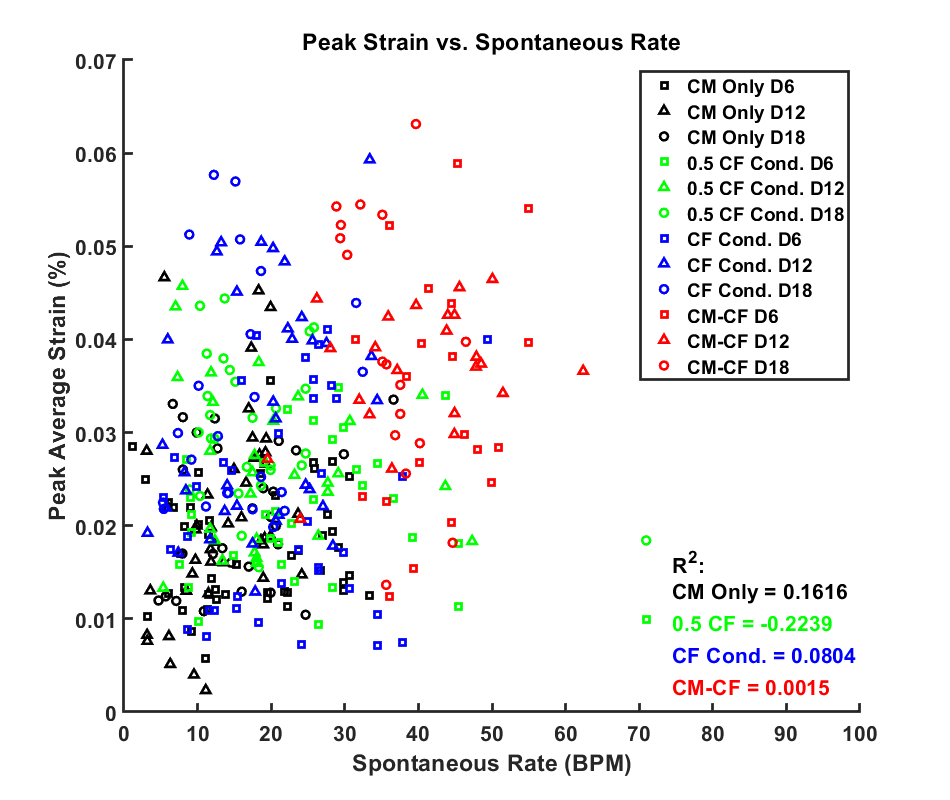
**

**Supplementary Figure 5.** No significant relationship exists between the maximum strain and the spontaneous rate of contraction. No correlation is found between the spontaneous rate (horizontal axis) and the peak average strain (vertical axis) for the CM Only (black), 0.5 CF Conditioned (green), CF Conditioned (blue), or CM-CF Coculture (red). R^2^ values were calculated using the full dataset for an experimental group (days 6, 12, and 18), though no significant linear correlation existed on any individual day for any group as well. All correlation p-values > 0.05.

## Supplementary Figure 6

**
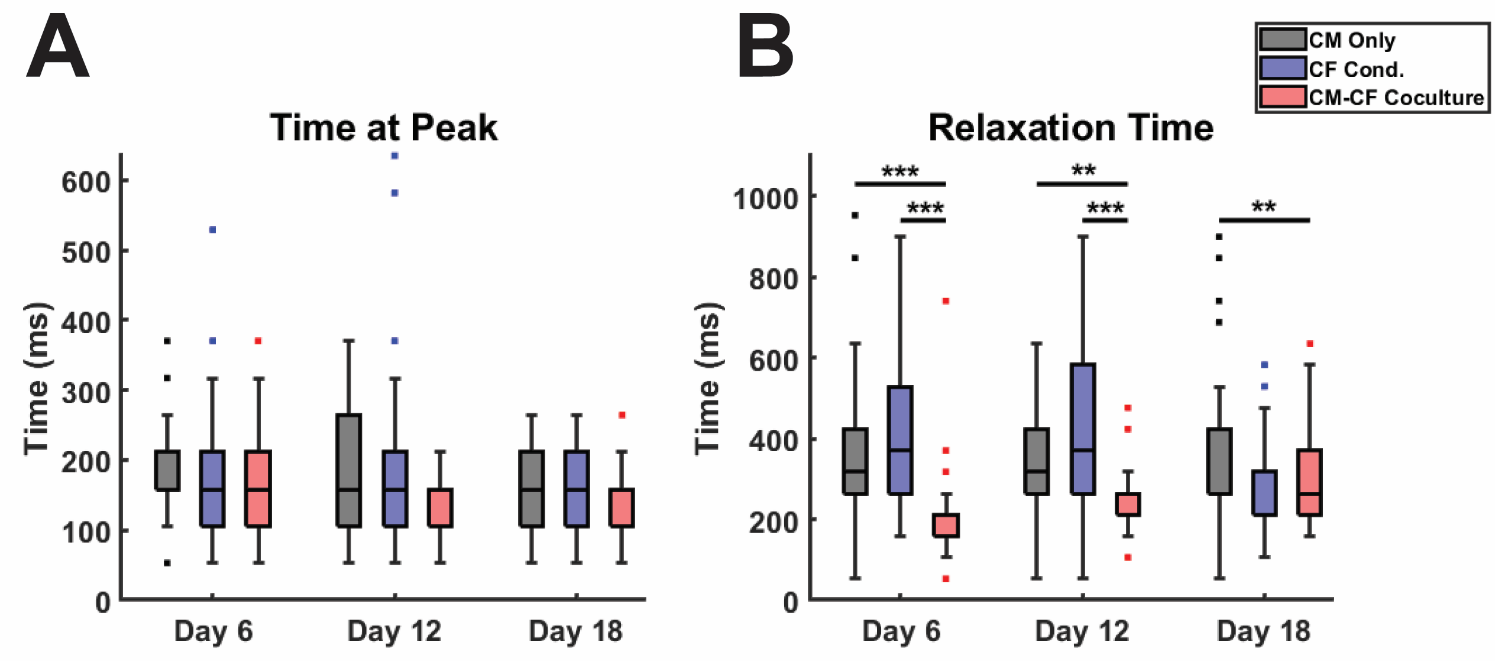
**

**Supplementary Figure 6.** Strain kinetics extended. (A) Time spent at the peak of contraction, or the time spent over the 90% strain threshold. (B) Relaxation time. N = CM Only: d6 = 47, d12 = 43, d18 = 28; CF Conditioned: d6 = 38, d12 = 35, d18 = 23; CM-CF Coculture: d6 = 20, d12 = 23, d18 = 17.

## Supplementary Figure 7


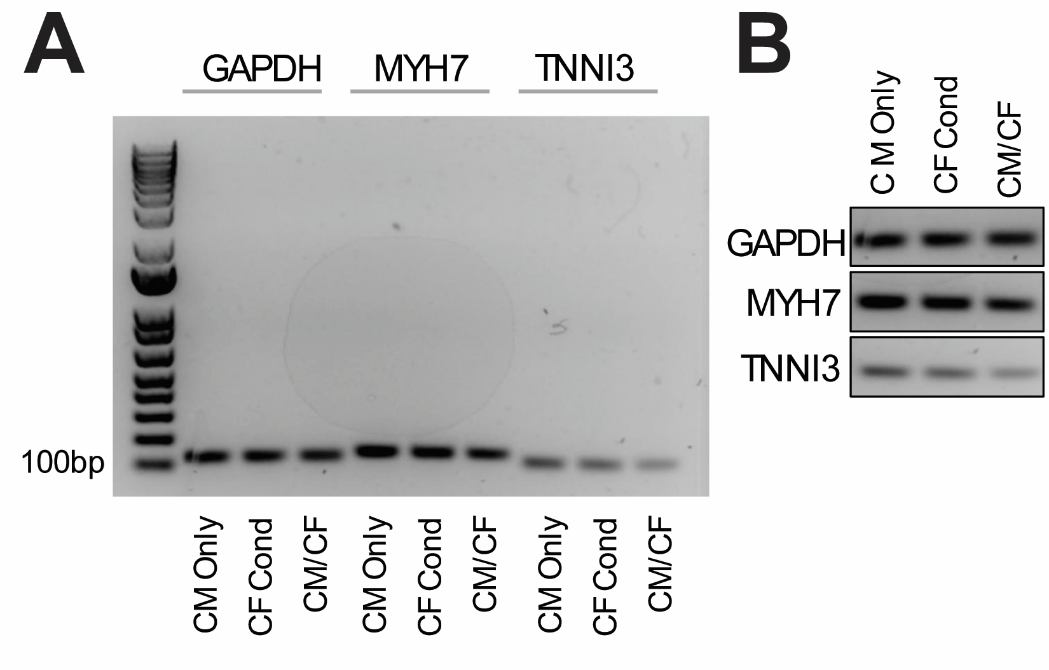


**Supplementary Figure 7:** Polymerase chain reaction (PCR) for sarcomeric genes associated with hiPSC-CM maturation. **(A)** Full PCR agarose gel with 1Kb+ DNA ladder displaying genes of interest at the correct band sizes: *GAPDH* (131bp), *MYH7* (148bp), *TNNI3* (118bp). **(B)** Cropped PCR gel demonstrating the expression of *MYH7* and *TNNI3* in all micropatterned conditions. No bands were present in no RT control (not shown). This technique is only to determine presence of maturation-associated mRNA/cDNA and is not quantitative.

## Supplementary Figure 8


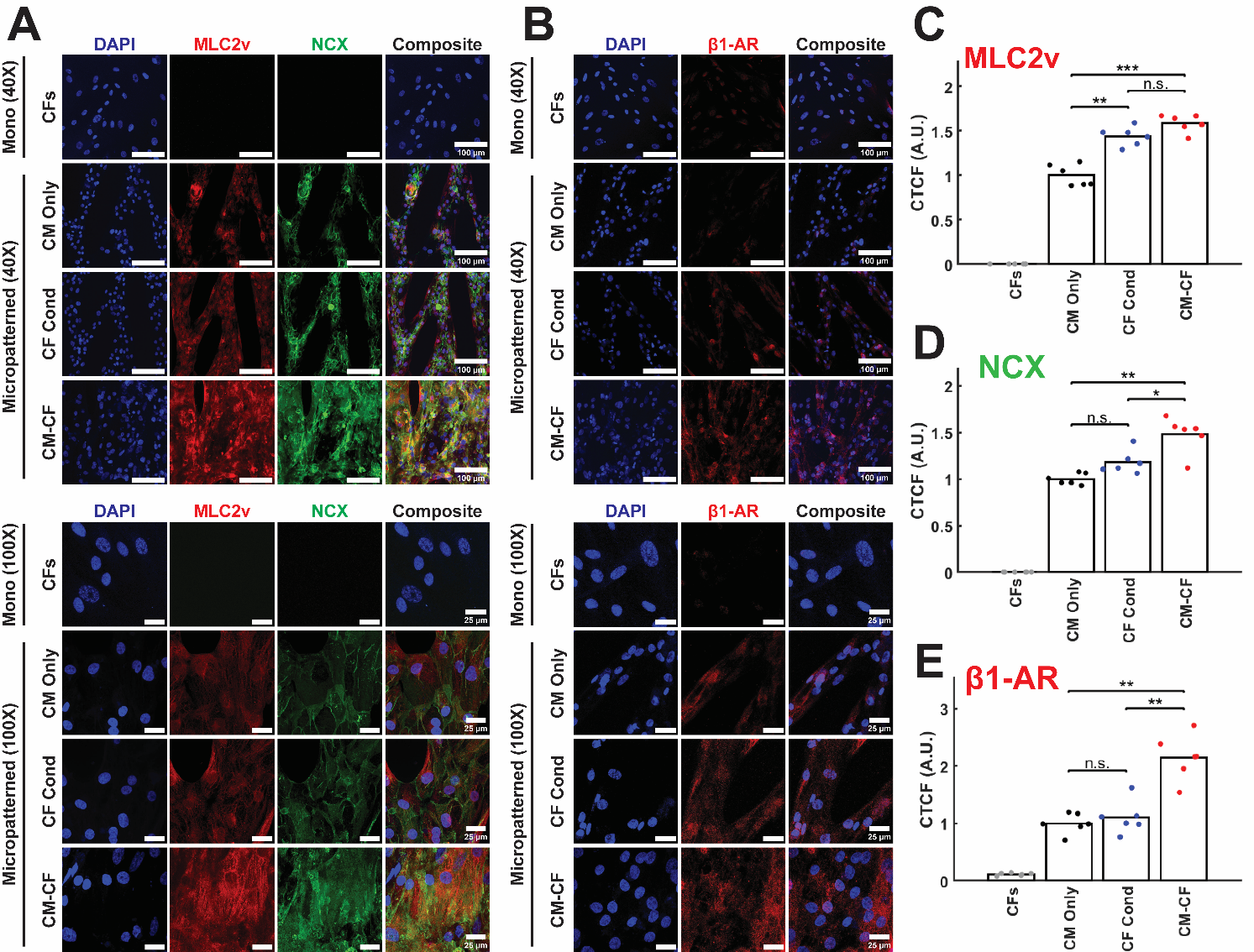


**Supplementary Figure 8.** Fluorescent quantification of immunolabeled iPSC-CM maturation markers. **(A)** Representative confocal microscopy images of monolayer iPSC-CFs, micropatterned CM Only, micropatterned CF Conditioned, and micropatterned CM-CF Coculture. Samples were stained for ventricular myosin light chain 2 (MLC2v, red), Na^+^/Ca^2+^ exchanger (NCX, green) and nuclei (DAPI, blue) and imaged at 40X (top, scale bars = 100 µm) and 100X (bottom, scale bars = 25 µm) magnification. **(B)** Representative confocal images of monolayer hiPSC-CFs, micropatterned CM Only, micropatterned CF Conditioned, and micropatterned CM-CF Coculture stained for beta-1 adrenergic receptors (β1-AR, red) and nuclei (DAPI, blue) and imaged at 40X (top, scale bars = 100 µm) and 100X (bottom, scale bars = 25 µm). **(C)** Quantification of MLC2v corrected total cell fluorescence (CTCF) for each sample group, normalized to the mean CTCF of CM Only. N = 6 (2 samples each, 3 locations per sample). **(D)** Quantification of NCX CTCF, normalized to the mean CTCF of CM Only. N = 6 (2 samples each, 3 locations per sample). **(E)** Quantification of β1-AR CTCF, normalized to the mean CTCF of CM Only. N = 6 (2 samples each, 3 locations per sample).

# Supplementary Tables

## Supplementary Table 1: PCR Primer Information

| Gene | For: | Rev: | Expected Size (bp) |
| --- | --- | --- | --- |
| *GAPDH* | GTCTCCTCTGACTTCAACAGC | ACCACCCTGTTGCTGTAGCCAA | 131 |
| *TNNI3* | CGTGTGGACAAGGTGGATGAAG | GCCGCTTAAACTTGCCTCGAAG | 118 |
| *MYH7* | GGAGTTCACACGCCTCAAAGAG | TCCTCAGCATCTGCCAGGTTGT | 148 |
